# Therapeutic Potential of Hydrogen Sulfide in Bone Deficiency

**DOI:** 10.3390/cimb48101029

**Published:** 2026-10-04

**Authors:** Ying Liu, Xuanming Zhang, Nana Cheng, Yunjia Song, Xutao Sun

**Affiliations:** 1 School of Basic Medical Sciences, Heilongjiang University of Chinese Medicine, Harbin 150040, China; ly15575154068@163.com (Y.L.); 18738982250@163.com (N.C.); 2 Department of Joint Surgery, The Second Affiliated Hospital of Harbin Medical University, Harbin 150086, China; zhangxuanming@hrbmu.edu.cn; 3 Department of Pharmacology, Heilongjiang University of Chinese Medicine, Harbin 150040, China

**Keywords:** hydrogen sulphide, bone deficiency, osteoporosis, drug use, bone metabolism

## Abstract

H_2_S, recognized as the third gaseous signaling molecule following NO and CO, holds significant potential for therapeutic applications in diseases characterized by bone deficiency. Considerable advancements have been achieved in the development of bone scaffold materials incorporating H_2_S donors, as well as in the creation of innovative H_2_S-releasing pharmacological agents. Exogenous supplementation of H_2_S has exhibited pronounced bone-protective effects in animal models of osteoporosis, fractures, and periodontitis. These therapeutic effects are mediated through various mechanisms, including anti-inflammatory and antioxidant activities, alongside the bidirectional regulation of bone metabolic balance. Mechanistically, H_2_S plays a critical role throughout the entire bone remodeling process—encompassing the resorption, reversal, and formation phases—by modulating osteoclast generation and activation, osteoblast differentiation and maturation, and maintaining mesenchymal stem cell function. This modulation occurs via key signaling pathways, such as the RANKL/OPG, the *Wnt*/β-catenin, and the p38-MAPK signaling pathway. A deeper understanding of the role of H_2_S in bone deficiency can lead to the discovery of new therapeutic targets, while innovative H_2_S donors offer highly promising new treatments for patients with bone deficiency.

## 1. Introduction

Bones serve as the body’s scaffold and mechanical support for movement; they are metabolically active and undergo continuous remodelling throughout a person’s lifetime. Bone remodelling serves both to adapt to mechanical loads and to facilitate the repair of bone damage, thereby maintaining skeletal integrity. The remodelling process is a continuous cycle that can be divided into three phases: the resorption phase, during which osteoclasts are recruited, activated, and carry out resorption on the surface of old or damaged bone; the reversal phase, during which osteoclasts undergo apoptosis and osteoblast precursor cells are recruited; and the formation phase, during which osteoblasts form new bone tissue [1]. Under steady-state conditions, there is a balance between bone resorption and bone formation. Under pathophysiological conditions (such as oxidative stress and inflammation), the homeostasis of bone tissue is disrupted; when bone resorption exceeds bone formation, bone deficiency occurs [2]. The most direct consequence of persistent bone deficiency is the development of systemic osteoporosis, which in turn increases bone fragility and the incidence of fractures. Localised bone deficiency, such as alveolar bone deficiency caused by periodontitis, may result in tooth mobility, abscesses or even tooth loss [3]. According to statistics, Europe incurs losses exceeding 56 billion euros annually due to osteoporosis and related fractures, amounting to approximately 4.3 million cases [4]. As of 2015, approximately 3.5 billion people worldwide were affected by oral diseases, with total global dental care expenditure reaching USD 544.41 billion—a figure that continues to rise [5]. These bone deficiency-related conditions not only significantly impair patients’ quality of life but also result in substantial economic losses, placing a heavy burden on healthcare systems. Currently, clinical guidelines in several European countries recommend oral bisphosphonates as the first-line treatment for osteoporosis [6]. However, these drugs may cause rare side effects and, as they cannot reverse the impaired bone formation capacity in osteoporosis patients, are often required to be used in combination with anabolic agents [7]. Non-surgical treatment of periodontitis further requires patients to undergo regular periodontal maintenance therapy [8]. Therefore, the search for new alternative treatments with distinct advantages is of great significance.

Hydrogen sulphide (H_2_S) was the third gaseous signaling molecule to be discovered, following nitric oxide (NO) and carbon monoxide (CO). In mammals, H_2_S is primarily produced by cystathionine-β-synthase (*CBS*), cystathionine-γ-lyase (*CSE*) and 3-mercaptopyruvate sulphurtransferase (*3-MPST*) [9]. Both *CBS* and *CSE* can directly produce H_2_S from L-cysteine and exhibit tissue specificity. *CSE*/H_2_S is predominant in peripheral organs [10]. It has been reported that the expression level of the *CSE* protein in osteoblasts isolated from the skulls of newborn rats is comparable to that in the liver, while the amount of H_2_S produced by *CBS* is only about one-third that of *CSE*, and *3-MPST* was not detected [11]. Real-time fluorescent probe analysis has demonstrated that mouse-derived osteoblasts also rely primarily on *CSE* for the direct production of H_2_S [12]. Endogenous H_2_S plays a role in regulating the self-renewal and osteogenic differentiation of bone marrow mesenchymal stem cells (BMMSCs); BMMSCs lacking H_2_S exhibit senescence and impaired osteogenic differentiation [13,14]. Mice with *CBS* knockout develop symptoms of osteoporosis due to reduced endogenous H_2_S levels [13]. Furthermore, H_2_S possesses anti-inflammatory and antioxidant properties, while inflammation and oxidative stress are the primary causes of bone deficiency induced by ageing and oestrogen deficiency [13,15]. Studies have shown that both NO and CO promote the osteogenic differentiation of BMMSCs and human periodontal ligament stem cells (PDLSCs) into osteoblasts, thereby facilitating bone regeneration [16,17,18,19]. Consequently, H_2_S holds immense potential for the treatment of bone deficiency-related conditions such as periodontitis, osteoporosis and fractures.

**Figure 1:**
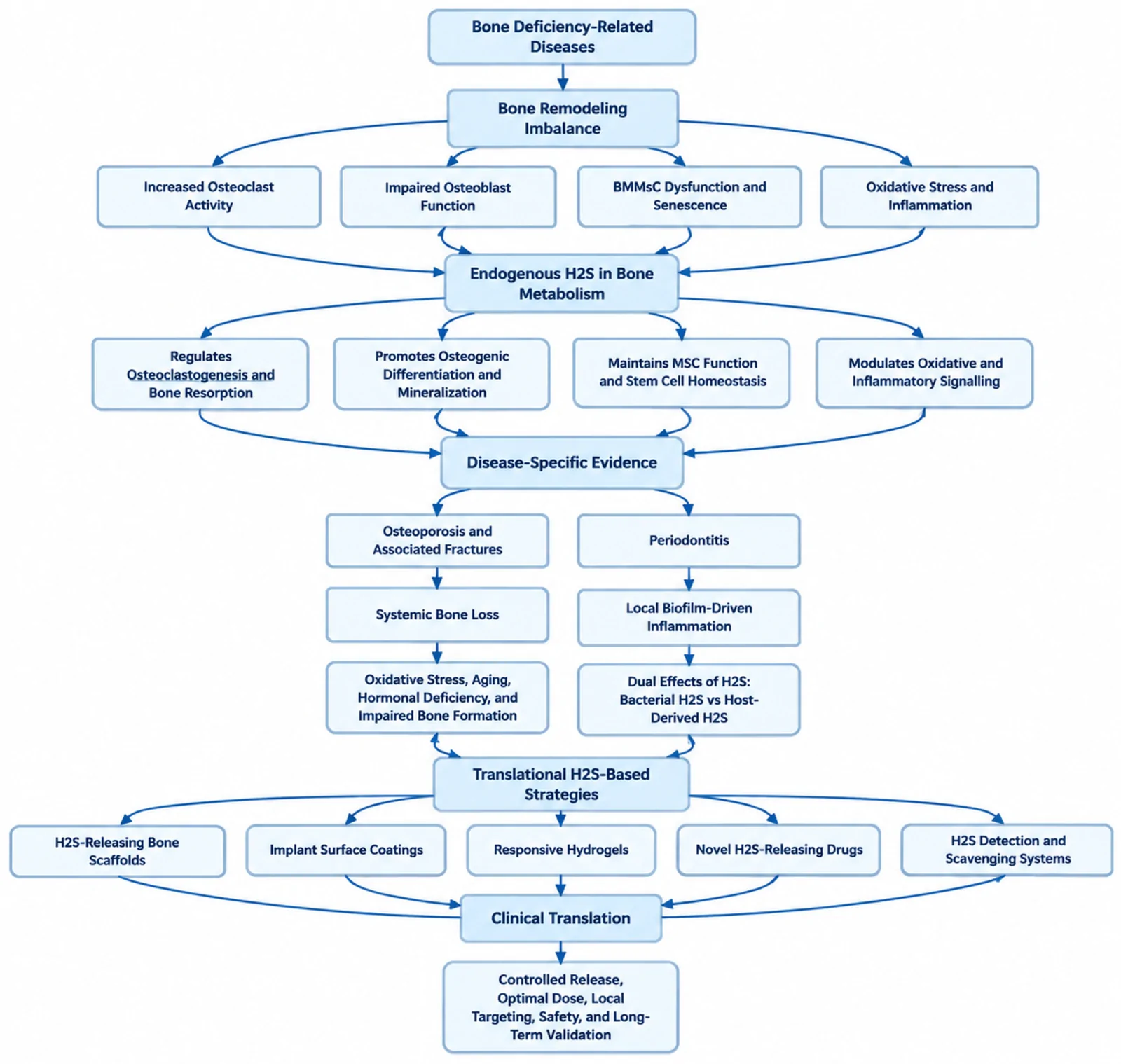
Overall framework of this review: from endogenous H_2_S-mediated bone remodeling to disease-specific mechanisms and translational H_2_S-based therapeutic strategies.

This article reviews the cellular and molecular mechanisms involved in the effects of H_2_S on periodontitis, osteoporosis, and related fractures, and summarizes existing and recently reported bone repair scaffolds as well as synthetic drugs that release H_2_S, with the aim of providing a theoretical foundation for the clinical translation of H_2_S. The overall framework of this review is shown in Figure 1 and Table 1.

## 2. Materials and Methods

### 2.1. Literature Search Strategy

A narrative literature review was conducted to summarize the roles of hydrogen sulfide (H_2_S) in bone metabolism, bone remodeling, bone regeneration, and bone loss-related diseases. Literature searches were performed in PubMed, Web of Science Core Collection, Scopus, and Google Scholar for articles published from 1 January 2009, to 31 December 2025. In addition, the reference lists of relevant original studies and review articles were manually screened to identify further eligible publications.

The search strategy combined H_2_S-related terms with terms related to bone cells, bone metabolism, bone diseases, and H_2_S-based therapeutic approaches. The main search terms were: (“hydrogen sulfide” OR “hydrogen sulphide” OR “H_2_S” OR “H2S”) AND (“bone” OR “bone metabolism” OR “bone remodeling” OR “bone remodelling” OR “bone regeneration” OR “osteogenesis” OR “osteoblast” OR “osteoclast” OR “mesenchymal stem cell” OR “bone marrow mesenchymal stem cell” OR “BMSC” OR “BMMSC” OR “periodontal ligament cell” OR “periodontal ligament stem cell” OR “PDLSC” OR “dental pulp stem cell” OR “DPSC” OR “osteoporosis” OR “periodontitis” OR “alveolar bone loss” OR “fracture healing” OR “bone defect” OR “bone tissue engineering” OR “scaffold” OR “implant”).

Additional searches were conducted using terms related to endogenous H_2_S-generating enzymes and signaling pathways, including “cystathionine-β-synthase” OR “*CBS*,” “cystathionine-γ-lyase” OR “*CSE*” OR “*CTH*,” “3-mercaptopyruvate sulfurtransferase” OR “*3-MPST*,” “H_2_S donor,” “H_2_S-releasing,” “GYY4137,” “NaHS,” “S-sulfhydration,” and “persulfidation.”

### 2.2. Study Selection

Original research articles were included if they investigated the effects or mechanisms of endogenous H_2_S, H_2_S-generating enzymes, H_2_S donors, H_2_S-releasing biomaterials, or H_2_S-targeted interventions in bone-related cells, bone metabolism, bone remodeling, bone regeneration, or bone loss-related diseases. Studies involving osteoblasts, osteoclasts, mesenchymal stem cells, periodontal ligament cells or stem cells, dental pulp stem cells, macrophages, chondrocytes, and relevant animal models were considered eligible when they reported outcomes related to osteogenic differentiation, osteoclastogenesis, bone loss, inflammation, oxidative stress, fracture healing, periodontal bone remodeling, bone regeneration, or osseointegration.

Articles were excluded when they were unrelated to bone biology, periodontal tissue, or bone tissue engineering; focused exclusively on H_2_S detection without a direct connection to bone-related disease or regeneration; lacked accessible full text; were conference abstracts, editorials, letters, or study protocols without experimental results; or were duplicate publications. Relevant review articles were used to identify additional references and provide background information but were not treated as primary mechanistic evidence.

## 3. Bone Metabolism

**Figure 2:**
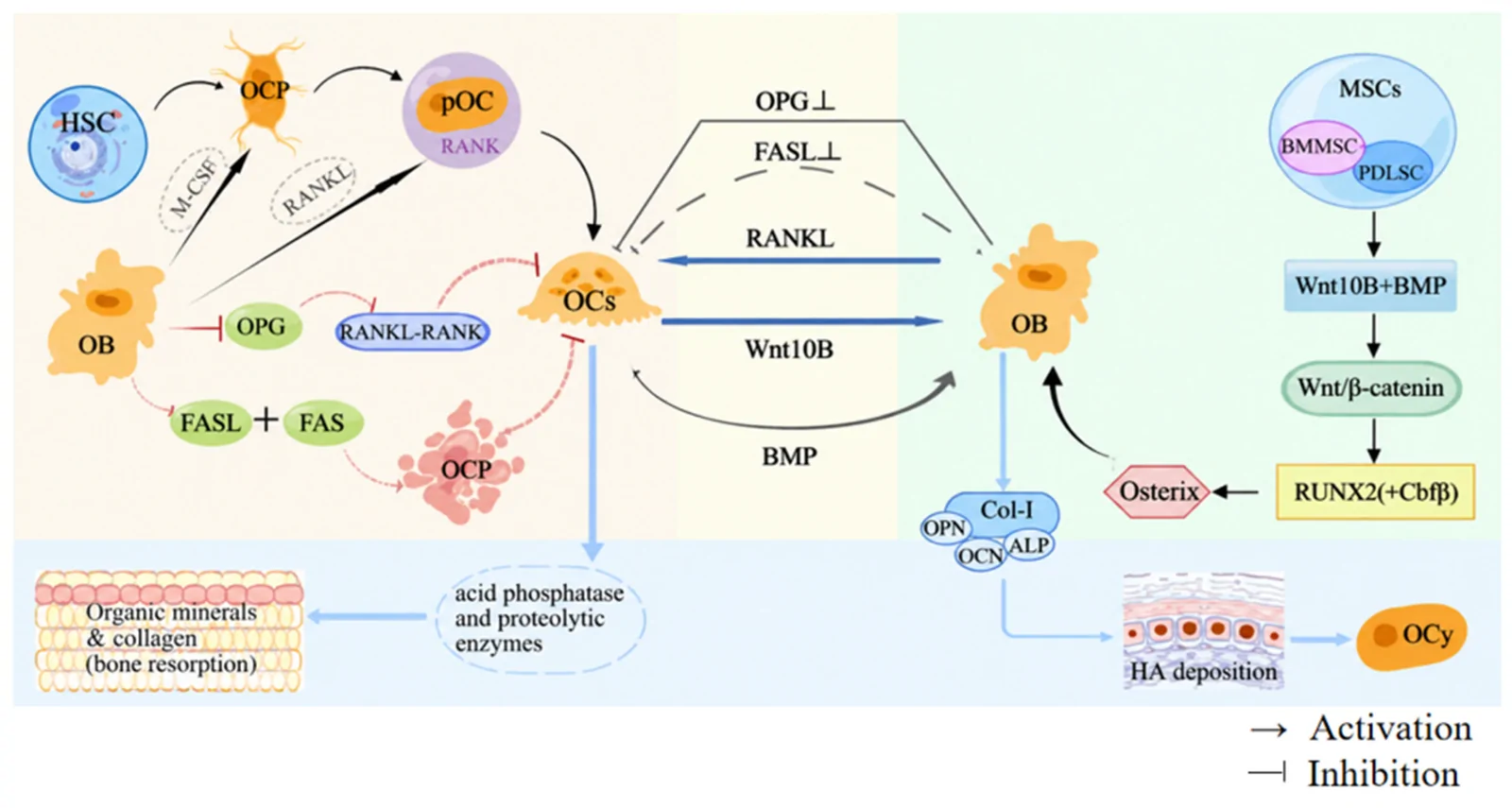
The reciprocal regulatory mechanisms between osteoclasts and osteoblasts in bone metabolic homeostasis. Created with BioGDP.com. 26 September 2026 [54].

The balance between bone resorption by osteoclasts and bone formation by osteoblasts maintains homeostasis in bone metabolism (Figure 2). Osteoclasts, which are multinucleated giant cells, originate from haematopoietic stem cells of the monocyte/macrophage lineage. Their differentiation process is governed by macrophage colony-stimulating factor (M-CSF) and receptor activator of nuclear factor κB ligand (RANKL), both of which are secreted by osteoblasts [72]. In the presence of M-CSF, osteoclast progenitor cells differentiate specifically into osteoclast precursors; subsequently, receptor activator of nuclear factor-κB (RANK) expressed on the surface of these precursors recognises RANKL on the osteoblast membrane via cell–cell interactions, thereby enabling multiple mononuclear osteoclast precursors to fuse and form mature multinucleated osteoclasts [73]. RANKL-RANK signaling continues to mediate the activation and survival of mature osteoclasts. Osteoclast differentiation is also negatively regulated by osteoprotegerin (OPG), produced by osteoblasts; this soluble decoy receptor inhibits osteoclast formation and bone resorption by competitively binding to RANKL in the presence of RANK [72]. Furthermore, osteoblasts can regulate the differentiation and survival of osteoclasts through direct cell-to-cell contact. For example, FAS ligand (*FASL*) expressed on the surface of osteoblasts is activated by oestrogen and subsequently binds to FAS receptors on osteoclast precursors, thereby inducing osteoclast apoptosis [74]. Upon activation, mature osteoclasts secrete acid phosphatase and proteases, thereby degrading the mineral and collagen matrix of bone tissue [73].

The differentiation of osteoblasts is also influenced by osteoclasts. Osteoblasts differentiate from mesenchymal stem cells (MSCs), which can be classified as BMMSCs and PDLSCs depending on their source [75]. Since MSCs are multipotent adult stem cells capable of differentiating into various cell types, the various factors influencing their specific differentiation pathways have become key regulators of the bone formation process, such as bone morphogenetic protein (BMP) and wingless-like protein 10B (*Wnt10B*) [76]. Mature osteoclasts are capable of releasing the early osteogenic regulator *Wnt10B* to promote the osteogenic differentiation of human MSCs [77]. The classical Wnt signaling pathway, which depends on β-catenin, is also referred to as the *Wnt*/β-catenin pathway. *Wnt10B* has been reported to promote osteoblast formation by inducing the expression of osteogenesis-related genes, including runt-related transcription factor 2 (*RUNX2*) and *Osterix* [78]. *RUNX2*, also known as core-binding factor α1 (Cbfa1), forms a heterodimer with the transcription factor core-binding factor subunit β (*Cbfβ*) and binds to DNA, thereby promoting the differentiation and maturation of MSCs into the osteoblast lineage [76]. *RUNX2* also directly regulates the *Wnt* signaling pathway; mice with *RUNX2* defects lack osteoblasts and are unable to form bone [79]. *Osterix* lies downstream of *RUNX2* and is activated by *RUNX2* to promote osteoblast differentiation; *Osterix*-knockout mice also fail to form bone [80]. Mature osteoblasts are surrounded by extracellular matrix proteins that they secrete themselves, including type I collagen, osteopontin, osteocalcin, and alkaline phosphatase (ALP). The osteoid formed by the hydration of this protein matrix is subsequently mineralised through the deposition of hydroxyapatite; ultimately, these osteoblasts differentiate into osteocytes that cover the bone surface [81].

## 4. Endogenous H_2_S Is Involved in the Regulation of Bone Metabolism

Endogenous H_2_S participates in multiple stages of bone metabolism, including osteoclastogenesis, bone resorption, osteoblast differentiation, and bone formation. During the resorption phase, H_2_S produced by periodontal ligament stem cells (PDLSCs) increases osteoclast activity in alveolar bone by activating the RANKL/OPG system and promoting monocyte chemoattractant protein-1 (MCP-1) secretion [82]. In addition, H_2_S enhances the survival and activity of mature osteoclasts by inhibiting autophagy and apoptosis through the phosphoinositide 3-kinase/protein kinase B/mechanistic target of rapamycin (PI3K/AKT/mTOR) pathway [83]. In orthodontic models, mechanical loading increases endogenous H_2_S signaling in PDLSCs, thereby activating the RANKL/OPG system and promoting root resorption and tooth movement [83,84]. Mechanical stimulation can also induce macrophage M1 polarization through signal transducer and activator of transcription 1 (STAT1), increasing osteoclast formation and further contributing to bone remodeling [85].

During the bone formation phase, H_2_S promotes the differentiation, maturation, and mineralization of osteoblast-lineage cells. Mechanical tension induces H_2_S production in periodontal ligament cells and increases the expression of alkaline phosphatase (ALP), osteocalcin, and type I collagen [86]. Under appropriate tensile stimulation, H_2_S promotes the osteogenic differentiation of human periodontal ligament cells through the p38 mitogen-activated protein kinase (p38-MAPK) pathway [87]. In dental pulp stem cells (DPSCs), H_2_S promotes transient receptor potential vanilloid 1 (*TRPV1*)-mediated calcium influx and activates β-catenin signaling, thereby supporting DPSC function and odontogenic differentiation [88]. In BMMSCs, endogenous H_2_S regulates calcium-channel sulfhydration and modulates PKC/ERK-dependent *Wnt*/β-catenin signaling, thereby maintaining the balance between self-renewal and osteogenic differentiation [13].

**Figure 3:**
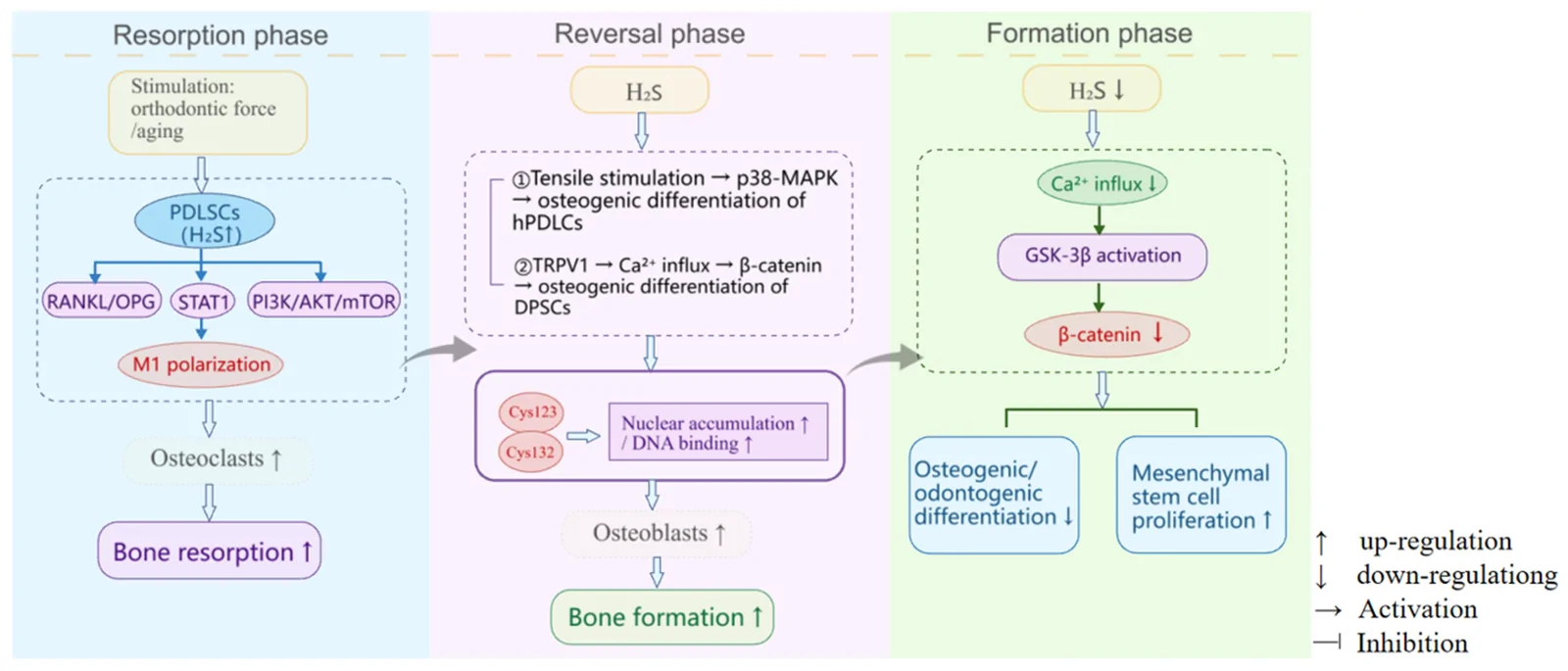
Triple regulatory mechanisms of endogenous H_2_S throughout the entire process of bone metabolism. Created with BioGDP.com. 26 September 2026 [54].

In osteoblasts, increasing endogenous H_2_S availability enhances *RUNX2* sulfhydration at Cys123 and Cys132, promotes its nuclear accumulation and DNA-binding activity, and thereby increases the transcription of osteogenic target genes [11]. Consistent with these findings, endogenous H_2_S promotes ALP and *RUNX2* expression, enhances ALP activity, and increases mineralized nodule formation in human mandibular BMMSCs [63]. Conversely, a reduction in intracellular H_2_S can alter calcium signaling and promote β-catenin degradation, thereby affecting the proliferation and differentiation of dental pulp mesenchymal cells [88]. Under both in vitro and in vivo conditions, H_2_S deficiency impairs the osteogenic and dentinogenic differentiation of DPSCs while promoting cell proliferation. This bidirectional regulation may provide a cellular reserve of osteogenic precursor cells and support the orderly progression of tissue repair after injury (Figure 3).

However, the precise mechanism by which H_2_S regulates the various stages of bone remodelling remains unclear. Studies in hPDLSCs have shown that excessive or insufficient H_2_S levels can impair osteogenesis by inhibiting *Wnt*/β-catenin signaling; an optimal concentration of endogenous H_2_S must be maintained to activate the *Wnt*/β-catenin signaling cascade and promote osteogenic differentiation [89]. This is because the signaling networks of osteoblasts during the bone formation phase (such as *Wnt* receptors and downstream kinases) have stricter ‘modification requirements’ for H_2_S; abnormal concentrations can lead to either insufficient or excessive modification, both of which block the pathway. H_2_S generated in Smooth muscle protein 22 alpha (*SM22α*) lineage niche cells via Platelet-derived growth factor receptor beta (*PDGFRβ*) promotion promotes osteogenesis and angiogenesis, while inhibiting the generation of hyperactive osteoclasts [90]. This suggests that osteoclast-related signaling (such as RANKL) during the bone resorption phase has a ‘narrow tolerance range’ for H_2_S concentrations, requiring the generation of ‘specific concentrations of H_2_S’ to achieve both the inhibition of ‘excessive osteoclast generation’ (thereby preventing excessive bone resorption) and the avoidance of interference with the basic requirements of bone resorption. The sensitivity of dental pulp mesenchymal cells (such as DPSCs) to H_2_S concentration manifests as a ‘response to even a slight decrease in concentration’ [88]. The signaling requirement during the reversal phase is to ‘rapidly activate the reserve for cell proliferation’, rather than fine-tuned differentiation regulation; consequently, cellular signaling pathways (such as Ca^2+^ channels) are more sensitive to ‘trends in H_2_S concentration’, and proliferation signals may be triggered as soon as the concentration falls below a threshold. In summary, the ‘functional objectives’ (resorption/remodelling/formation) at different stages determine the ‘signaling requirements’ of the core cells, and these differing requirements directly result in varying ‘sensitivity thresholds’ to H_2_S concentrations. This difference enables H_2_S to precisely switch its regulatory direction in accordance with the dynamic process of bone remodelling, ultimately achieving orderly regulation of bone remodelling. This also explains why different cell types (hPDLSCs, *SM22α* niche cells, DPSCs) exhibit varying responses to H_2_S.

## 5. The Therapeutic Potential of H_2_S for Diseases Involving Bone Deficiency

### 5.1. Osteoporosis and Associated Fractures

Osteoporosis is a systemic skeletal disorder characterised primarily by reduced bone strength. Its onset is directly related to systemic bone deficiency, which can be either physiological or pathological in nature. Physiological bone deficiency typically occurs alongside ageing and natural fluctuations in hormone levels, and progresses relatively gradually; pathological bone deficiency, on the other hand, is triggered by underlying diseases, side effects of medications, or severe nutritional deficiencies, among other factors. It results in a faster rate of bone deficiency and a significantly higher risk of fractures. As the most serious complication of osteoporosis, the occurrence of osteoporotic fractures is closely linked to a decline in bone density, deterioration in bone quality, and damage to bone microstructure. At this stage, bone fragility increases significantly; even minor everyday stresses such as coughing, bending over, or a slight fall may trigger a fracture. Such fractures not only have a prolonged healing period and are subject to significant variations in healing quality due to the disease, but also tend to impede the restoration of bone function, while substantially increasing the patient’s risk of subsequent refractures. From a pathophysiological perspective, the elderly population is highly susceptible to osteoporosis, with the onset of the disease driven by a combination of multiple mechanisms: ageing of BMMSCs leads to a reduction in osteoblast supply; oxidative stress directly impairs osteoblast function and weakens bone formation capacity; chronic inflammation exacerbates osteoclast activation; sex hormone deficiency disrupts the RANKL-OPG balance; insufficient mechanical stress reduces bone formation signaling; and vitamin D deficiency causes impaired calcium absorption. The combined effect of these factors leads to an imbalance in bone metabolism, ultimately resulting in osteoporosis. By regulating several of these key mechanisms, H_2_S can help maintain the balance of bone metabolism, thereby providing an important direction for research into the disease’s mechanisms and potential interventions.

H_2_S availability and endogenous H_2_S-generating activity in BMMSCs decline with age, whereas inhibition of H_2_S production accelerates BMMSC senescence. In vitro supplementation with H_2_S restores BMMSC function, and in vivo H_2_S treatment attenuates bone loss. Mechanistically, H_2_S inhibits cellular senescence through the calcineurin-NFAT1 signaling pathway [14] and maintains the balance between BMMSC self-renewal and osteogenic differentiation by regulating *Wnt*/β-catenin signaling [13]. These findings suggest that H_2_S supplementation may reverse the depletion of the stem cell pool caused by physiological ageing of BMMSCs, providing sustained stem cell support for bone homeostasis. Furthermore, H_2_S enhances the activity of Sirtuin 3 (*SIRT3*) by S-sulfhydrating the Cys-X-X-Cys (CXXC) site of its zinc-finger motif, thereby reversing oxidative stress-induced senescence in BMMSCs—a process characterised by mitochondrial dysfunction and the accelerated dissociation of the heterochromatin protein Trimethylation of histone H3 at lysine 9 (H3K9me3) from the nuclear membrane protein Lamin B1 [91]. This is primarily related to the antioxidant effects of H_2_S. Studies have shown that H_2_S treatment may alleviate bone deficiency below the lesion induced by complete spinal cord injury by reducing oxidative stress, inhibiting Matrix metalloproteinase (MMP) activity, and activating *Wnt*/β-catenin signaling [37]. In an H_2_O_2_-induced oxidative injury model, H_2_S protected MC3T3-E1 osteoblasts from cellular damage, and this effect was associated with the regulation of MAPK (p38 and ERK1/2) signaling [20]. In particular, about ERK1/2, both the rapidly released H_2_S donor Sodium hydrosulfide (NaHS) and the sustained-release donor GYY4137 can inhibit this pathway to alleviate oxidative stress, while simultaneously enhancing the transcription and activity of ALP in osteoblasts to stimulate osteoblast proliferation [20,25,37]. In addition, NaHS treatment stimulated the transcriptional levels of osteocalcin, as well as the protein expression of collagen, a major component of bone tissue. It is worth noting that oxidative stress not only reduces osteogenesis but also promotes osteoclastogenesis. However, current research has primarily focused on how H_2_S reduces damage to osteoblasts to counteract osteoporosis, while attention to its effects on osteoclasts remains insufficient. There is an interplay between oxidative stress and chronic inflammation; both are considered intrinsic mechanisms leading to osteoporosis and can increase osteoclast activity. Macrophage polarisation is one of the core mechanisms by which the body precisely regulates inflammatory responses; its different polarisation phenotypes directly determine whether inflammation is ‘initiated and amplified’ (M1 polarisation) or ‘resolved and repaired’ (M2 polarisation), with the two forming a dynamic equilibrium that jointly maintains tissue homeostasis. H_2_S can both induce M1 polarisation of macrophages, thereby amplifying inflammation, and the resulting increase in osteoclast numbers [85]; it can also modify M2 macrophage-derived exosomes to promote their endocytosis by MSCs, thereby promoting the osteogenic differentiation of MSCs [26]. During HHcy in mice, H_2_S epigenetically attenuates bone deficiency through OPG/RANKL regulation [64]. Under HHcy conditions, c-Jun N-terminal kinase (JNK) binds to the *DNMT1* promoter following phosphorylation and activation; the subsequent upregulation of *DNMT1* leads to epigenetic hypermethylation of the OPG gene, which in turn activates RANKL expression and mediates osteoclastogenesis; however, H_2_S can reverse this outcome. The acetylated lysine residue, acetylation of histone H3 at lysine 27 (H3K27ac) of the nuclear chromatin histone, binds to the promoters of inflammatory factors (Interleukin-6 (IL-6), Tumor necrosis factor-α (TNF-α)), thereby promoting their transcriptional activation. HHcy inhibits the activity of histone deacetylase 3 (*HDAC3*), while H_2_S modifies *RUNX2* via thiolation. H_2_S balances the key epigenetic regulator *HDAC3* and the inflammatory NF-κB signaling pathway under HHcy conditions observed in BMMSCs from *CBS*-deficient mice [30]. HHcy reduces intracellular H_2_S and *CBS*/*CSE* levels; it can induce mitochondrial toxicity and apoptosis, while NaHS treatment reduces the release of cytochrome C from mitochondria into the cytoplasm. H_2_S restored ATP production, mitochondrial DNA copy number, and oxygen consumption in osteoblasts, indicating a protective effect against HHcy-induced mitochondrial toxicity [31]. Therefore, future research into H_2_S regulation of osteoporosis should move beyond the current focus on osteoblast protection and shift towards investigating its effects on osteoclast function and exploring bidirectional osteoblastic-osteoclastic regulatory mechanisms.

**Figure 4:**
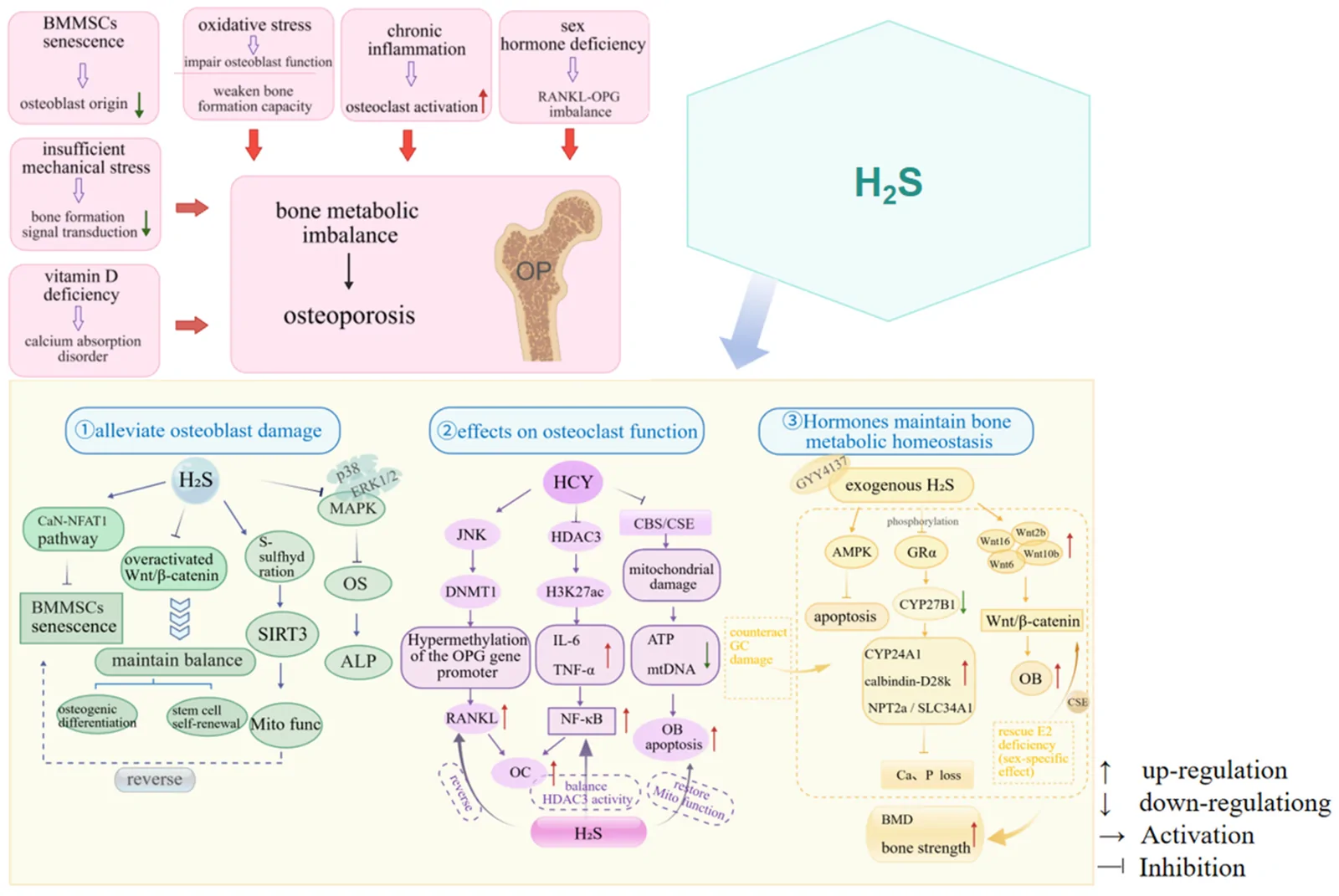
H_2_S ameliorates osteoporosis through multi-target regulatory mechanisms. Created with BioGDP.com. 26 September 2026 [54].

Hormones are key regulatory factors in maintaining the balance of bone metabolism; exogenous H_2_S has been shown to reliably increase bone density and improve bone strength in osteoporosis models induced by various factors, such as glucocorticoids and menopause. H_2_S inhibits dexamethasone-induced decreases in osteoblast activity, cell death and apoptosis by activating the AMP-activated protein kinase (AMPK) signaling pathway [92]. And maintains the balance between BMMSC self-renewal and osteogenic differentiation by regulating *Wnt*/β-catenin signaling [21]. In contrast, blocking the *Wnt*/β-catenin signaling pathway significantly attenuated the effects of H_2_S on osteoblasts. Following fracture, serum H_2_S levels decrease while glucocorticoid levels increase. Supplementation with GYY4137 reduces glucocorticoid secretion, inhibits glucocorticoid receptor α activity through sulfhydration, downregulates vitamin D 1α-hydroxylase, and upregulates renal 24-hydroxylase, calbindin-D28k, and sodium phosphate cotransporter 2a, thereby inhibiting fracture-induced calcium and phosphorus loss [33]. H_2_S is a novel regulator of bone formation, implicated in oestrogen-deficient bone deficiency [34]. H_2_S activates *Wnt* signaling by increasing the production of the *Wnt* ligands *Wnt16*, *Wnt2b*, *Wnt6*, and *Wnt10b* in the bone marrow, thereby enhancing osteoblast formation in mice. The *CSE*/H_2_S system is associated with oestrogen signaling in bone in a sex-specific manner. Oestrogen receptors bind to the *CSE* promoter region to upregulate *CSE* protein expression in human osteoblasts, and this effect is sex-specific [23]. Following ovariectomy, bone mineral density, serum calcium, serum phosphate, and calcitonin all decrease, while parathyroid hormone and leptin increase; the results in the GYY4137 treatment group were the opposite [24]. Exogenous H_2_S exerts a modulatory effect on osteoporosis in ovariectomized rats and holds potential value for the treatment of postmenopausal osteoporosis in humans. Furthermore, H_2_S donors, serum obtained during cinacalcet treatment, and serum collected before and after haemodialysis were separately evaluated for their effects on the osteogenic potential of mesenchymal stem cells under uremic conditions. These experimental conditions improved selected osteogenic and mineralization-related parameters; however, the study did not investigate a combined cinacalcet-plus-haemodialysis treatment protocol, and therefore did not establish a synergistic effect between these interventions [29]. Uremic toxins in serum lead to a decrease in the RANKL/OPG ratio in human mesenchymal stem cells (hMSCs). Bone remodeling in uremia is influenced by the ability of the uremic environment to alter the osteogenic differentiation of hMSCs (Figure 4).

Although osteoporosis and fracture healing are primarily systemic skeletal conditions, H_2_S-related bone deficiency is not restricted to the appendicular skeleton. Similar disturbances in osteoblast-osteoclast coupling, oxidative stress, inflammation, and impaired regeneration also occur in the alveolar bone. However, unlike osteoporosis, periodontitis is driven by a local microbial biofilm and is characterized by the coexistence of bacterially derived H_2_S and host-derived H_2_S. This distinct microenvironment makes the role of H_2_S in periodontitis more complex and necessitates separate consideration of its pathogenic and protective actions.

### 5.2. Periodontitis

**Figure 5:**
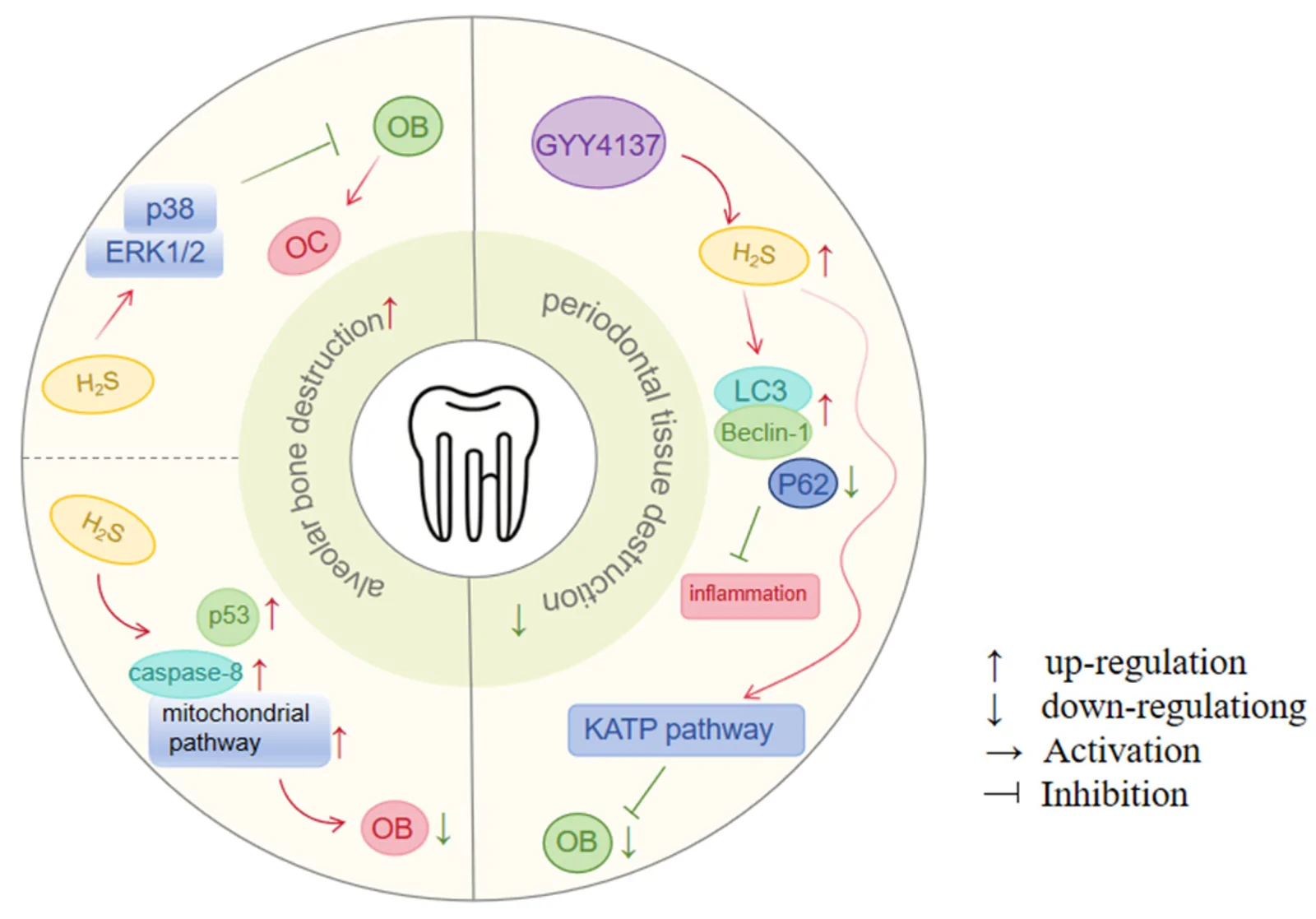
The Dual Regulatory Role of H_2_S in Periodontitis and Its Potential Mechanism in Diabetes-Associated Periodontitis. Created with BioGDP.com. 26 September 2026 [54].

Periodontitis is a chronic, destructive inflammation of the periodontal tissues caused by dental plaque biofilm. It is one of the leading causes of tooth deficiency in adults, has a high global prevalence, and is closely linked to overall health. The core aetiological factor is dental plaque—a bacterial colony on the tooth surface and within the gingival sulcus. If not thoroughly cleaned over a prolonged period, dental plaque gradually mineralises to form tartar, which continuously irritates the gums and invades deeper periodontal tissues, damaging the gums, periodontal ligament, alveolar bone and cementum, ultimately leading to gum recession, exposed tooth roots, tooth mobility and even tooth deficiency. Furthermore, factors such as smoking, diabetes, chronic psychological stress, malnutrition (e.g., vitamin C deficiency) and genetic predisposition can reduce the body’s resistance to inflammation, thereby exacerbating the progression of periodontitis. Conversely, periodontitis may also adversely affect general health, making diabetes more difficult to manage and increasing the risk of cardiovascular disease; periodontitis during pregnancy may also be associated with preterm birth and low birth weight. H_2_S is a key metabolite produced by pathogenic bacteria in the periodontal pocket through the metabolism of sulphur-containing amino acids (such as cysteine). Studies have shown that, in rat models, the administration of H_2_S alone induces transient osteoclast differentiation via RANKL expression, thereby promoting the differentiation and maturation of osteoclasts [22]. H_2_S activates ERK1/2 and p38 by promoting their phosphorylation, thereby inhibiting the proliferation of human osteoblasts while promoting osteoclast proliferation [40,41]. When present at sub-pathological concentrations in gingival crevicular fluid, H_2_S triggers osteoblast apoptosis via a molecular cascade involving Tumor protein p53, mitochondrial signaling, and caspase-8 activation [42]. However, a study in rats showed that although ligation promoted alveolar bone deficiency, H_2_S application did not exacerbate this effect and concurrently attenuated inflammation [43]. Local administration of GYY4137 further aggravated both periodontal inflammation and alveolar bone deficiency induced by ligature placement in rats [48]. Although GYY4137 aggravates periodontal inflammation and alveolar bone deficiency, it concurrently exerts a protective effect by activating autophagy in human periodontal ligament cells, as evidenced by upregulated Microtubule-associated protein 1A/1B-light chain 3 (LC3), Beclin-1, and downregulated Sequestosome-1 (p62). A high-glucose environment stimulates an increase in the concentration of lipopolysaccharide (LPS) from *Porphyromonas gingivalis* and the expression of *CSE* in periodontal ligament cells; knocking out *CSE* inhibits the phosphorylation and activation of the nuclear factor-kappa B p65 subunit (NF-κB p65), thereby reducing the expression of pro-inflammatory cytokines. Concurrently, experimental periodontitis in mice led to diminished bone deficiency, decreased leukocyte infiltration, and impaired osteoclast formation [49]. H_2_S protects primary osteoblasts from high-glucose-induced inhibition of osteoblast proliferation and apoptosis by upregulating ATP-sensitive potassium channels (KATP). H_2_S exhibits a dual nature in periodontitis pathogenesis: it is generated from sulfur-containing amino acid metabolism by periodontal pathogens, while simultaneously acting as a modulator of bone turnover. Evidence indicates that H_2_S upregulates RANKL expression, thereby facilitating osteoclast differentiation and maturation; by activating ERK1/2 and p38, it inhibits osteoblast proliferation while promoting osteoclast proliferation; at concentrations below pathological levels, it also induces osteoblast apoptosis (involving p53, the mitochondrial pathway and caspase-8 activation). Contradictorily, however, in rat experiments, H_2_S did not exacerbate ligature-induced alveolar bone deficiency and was able to alleviate inflammation; while topical application of GYY4137 exacerbated periodontal inflammation and bone deficiency, it could suppress excessive inflammation by activating autophagy in periodontal ligament cells. Of particular note is that, as diabetes is a risk factor for periodontitis, a high-glucose environment elevates the concentration of LPS from *Porphyromonas gingivalis* and the expression of *CSE* in periodontal ligament cells; knocking out *CSE* inhibits NF-κB p65 phosphorylation, reduces the release of pro-inflammatory factors and alleviates bone ldeficiency; at the same time, H_2_S can protect primary osteoblasts from hyperglycaemia-induced proliferation inhibition and apoptosis by upregulating KATP channels. This implies that, in diabetes-associated periodontitis, H_2_S may regulate KATP channels to balance osteocyte function under hyperglycaemic conditions and limit periodontal tissue destruction, thus offering a novel therapeutic avenue for this complication (Figure 5).

Given the insidious nature of periodontal tissue destruction, early diagnosis is crucial for periodontitis. H_2_S in saliva has been identified as a potential biomarker for monitoring the progression of periodontitis. However, existing detection methods struggle to precisely locate the specific sources of H_2_S release within the oral cavity, making it difficult to accurately identify affected areas and significantly limiting the early diagnosis and precise treatment of periodontitis. To address this technical gap, researchers have developed several novel detection probes and related technological platforms. Among these is a wearable hydrogel-based radiofrequency (RF) sensor capable of in situ H_2_S detection while simultaneously providing antimicrobial therapy [52]. This RF sensor integrates an agarose hydrogel with a split-ring resonator; the hydrogel is loaded with silver nanoparticles and chlorhexidine (AG-AgNPs-CHL). When applied to the tooth surface, this hydrogel-based RF sensor can wirelessly transmit sensing signals to a mobile device while simultaneously releasing chlorhexidine—which possesses broad-spectrum antibacterial activity—without the need for complex circuitry. Thanks to the selective binding mechanism between AgNPs and thiols, the sensor exhibits good sensitivity, a wide detection range (2–30 μM), and a low detection limit (1.2 μM). Meanwhile, by loading iron-doped molybdenum oxide with oxygen vacancies (MoO_3−x_) onto Titanium dioxide (TiO_2_) nanotubes, a high-performance metal-oxide-semiconductor (MOS)-based chemoresistive H_2_S sensor was developed [56]. In this composite system, the substitution of Fe for Mo in MoO_3−x_ not only creates oxygen vacancies but also introduces defect levels into the MoO_3−x_/TiO_2_ heterostructure, which jointly promote the gas-sensing reaction under ambient conditions (room temperature and normal illumination). The as-prepared sensor offers ultra-high sensitivity, swift response and recovery, and a wide dynamic response range for H_2_S concentrations as high as 400 ppm [57]. Specifically, the response amplitudes for 10 ppm and 100 ppm H_2_S were 1.75–5.66 and 5.56–12.13, respectively, and the sensor exhibited unique electronic response characteristics, enabling it to effectively distinguish target gases within complex gas mixtures. Furthermore, a novel near-infrared (NIR) light-enhanced colorimetric platform has been developed for the ultra-sensitive detection of disease-related volatile sulphides (VSCs), including H_2_S in the oral cavity, the concentration levels of which can be used to assess the severity of various periodontal diseases [58]. The detection range of this platform covers VSC concentrations in patients with varying degrees of periodontitis under pathophysiological conditions. Furthermore, in conjunction with smartphone-assisted colourimetric analysis, non-invasive, real-time monitoring of VSC levels can be achieved simply by taking a photograph of a saliva sample. In addition, researchers have developed an integrated heterostructure composite material based on two-dimensional Molybdenum carbide MXene (Mo_2_CTx)-modified Molybdenum diselenide (MoSe_2_), which can be used for H_2_S detection at room temperature and has been applied to the diagnosis of periodontitis [59]. Real-time breath analysis further confirmed that this sensor is capable of dynamically tracking trace amounts of H_2_S and that monitoring H_2_S levels can effectively distinguish between healthy individuals and patients with periodontitis. Another study developed a local surface plasmon resonance (LSPR) sensor for H_2_S detection, fabricated by coating a gold nanopatterning surface with a zinc oxide (ZnO) film, which is capable of measuring the cumulative concentration of H_2_S. The sensitivity of this sensor increases with rising relative humidity; at 70% relative humidity, it can detect H_2_S concentrations ranging from 0.05 to 3 ppm within 10 minutes, while showing no response to methyl mercaptan or dimethyl sulphide, demonstrating excellent selectivity [60]. It exhibits high sensitivity and rapid response to H_2_S, while simultaneously outputting dual signals in the form of colour and fluorescence changes, enabling efficient real-time monitoring of H_2_S [61]. Furthermore, the surface film of this sensor can be used for the real-time diagnosis of periodontitis, accurately identifying affected tooth areas through visible colour and fluorescence changes. In addition, the film exhibits a high capacity for reactive oxygen species (ROS) generation, which can effectively inhibit the growth of periodontal pathogens, thereby achieving an integrated design combining diagnostic and therapeutic functions.

Various diagnostic probes have established a correlation between the concentration and distribution of H_2_S and the severity and location of periodontitis. However, this does not imply that H_2_S lacks therapeutic potential for periodontitis. Although some current research is focused on reducing localised H_2_S levels in the periodontal tissues to alleviate inflammation, it must be made clear that H_2_S itself is not the primary cause of periodontal inflammation—the core pathogenic drivers of periodontitis are pathogenic bacteria (such as *Porphyromonas gingivalis*) within the dental plaque biofilm and their toxic by-products, among which bacterial LPS is a key factor in initiating and exacerbating periodontal inflammation, while H_2_S produced by bacterial metabolism is primarily associated with halitosis. It is worth noting that studies have confirmed that when H_2_S coexists with LPS, a synergistic effect occurs, triggering a stronger inflammatory response than when either acts alone, and it also exerts an additive effect on osteoclast differentiation [62], suggesting that the accumulation of locally produced bacterial H_2_S in the periodontium may have an ‘amplifying association’ with the toxic effects of LPS: when H_2_S concentrations reach a certain threshold, it may exacerbate LPS-mediated damage to periodontal tissues by increasing cellular sensitivity to LPS or by activating shared inflammatory signaling pathways (such as the NF-κB pathway). In vitro studies have shown that H_2_S levels in osteoblasts increase upon LPS stimulation [12]. On the other hand, endogenous H_2_S (synthesised by resident cells such as PDLSCs and gingival fibroblasts) exhibits clear anti-inflammatory and tissue-protective effects at appropriate concentrations. NaHS can block the LPS- and endotoxin-induced activation of p38, ERK, MAP kinase phosphatase-1 (MKP-1), PI3K, PKC, PKC isoenzymes and NF-κB, while inducing osteoblast differentiation and inhibiting osteoclast differentiation [44]. S-propynylcysteine increases *CSE* levels in gingival tissue and H_2_S levels in peripheral blood; by inhibiting the ERK/cAMP response element-binding protein (CREB) signaling pathway, it modulates the Th17/Treg balance, thereby preventing the progression of periodontitis [45]. Therefore, rather than indiscriminately eliminating local H_2_S in the periodontium, dynamically monitoring and regulating H_2_S levels within the ‘optimal anti-inflammatory protective range’ (i.e., suppressing the inflammatory amplification effect of bacterially derived H_2_S while preserving the reparative function of endogenous H_2_S) using detection probes may represent a more precise therapeutic strategy for periodontitis.

Compared with simply suppressing inflammation to alleviate acute damage to periodontal tissues, the unique osteogenic effects of H_2_S—such as promoting the osteogenic differentiation of PDLSCs and accelerating alveolar bone repair—hold greater significance in repairing inflammation-related periodontal bone deficiency, thereby offering a new dual-target approach of ‘anti-inflammatory + repair’ for the treatment of periodontitis. However, the application of H_2_S regulation in the clinical treatment of periodontitis still requires in-depth consideration of the following key scientific issues and technical bottlenecks: Firstly, in previous studies, H_2_S was predominantly administered via intraperitoneal injection to counteract the pro-inflammatory effects of LPSs; the correlation between this route of administration and blood drug concentrations needs to be revalidated in periodontal disease models. As periodontitis is a localised chronic infectious disease with lesions confined to the periodontal pocket and surrounding soft and hard tissues, it remains unclear whether H_2_S in the blood following intraperitoneal administration can effectively accumulate at the periodontal site, whether its local concentration reaches the pharmacological threshold required for anti-inflammatory effects, and whether there is a linear correlation with systemic blood concentrations. The local periodontal microenvironment (e.g., hypoxia, acidity, high levels of bacterial toxins) may alter the stability and efficacy of H_2_S; consequently, H_2_S concentration standards established under systemic administration cannot be directly equated with the effective concentrations required for local anti-inflammatory action in the periodontium. It is therefore necessary to establish a local delivery system and concentration assessment criteria specific to periodontal disease models. Secondly, the sources and concentration characteristics of H_2_S in periodontal disease are unique, necessitating a precise distinction between endogenous synthesis and exogenous production. On the one hand, studies at the bacterial level have confirmed that periodontal pathogens (such as *Porphyromonas gingivalis* and *Fusobacterium foersterii*) can generate exogenous H_2_S through the metabolism of sulphur-containing amino acids, with its concentration showing a positive correlation with bacterial load; on the other hand, studies at the host cell level have shown that LPS can upregulate the expression of *CSE* in osteoblasts and PDLSCs, thereby promoting endogenous H_2_S synthesis; This implies that, in periodontal disease, H_2_S concentrations result from the combined effects of ‘exogenous bacterial production’ and ‘endogenous compensatory synthesis by the host’, representing a significant difference from the concentration profile observed during normal periodontal tissue remodelling, which is ‘dominated by endogenous H_2_S from the host’. Consequently, future research must clarify the ratio of endogenous to exogenous H_2_S at different stages of the disease (such as gingivitis and mild, moderate and severe periodontitis) to avoid the dual problems of ‘insufficient suppression of pathogenic bacterial H_2_S’ or ‘interference with the host’s compensatory H_2_S synthesis’ that may arise from interventions using a fixed concentration of H_2_S. Furthermore, the precise regulation and real-time detection of endogenous H_2_S face technical bottlenecks. On the one hand, there is currently a lack of effective local regulatory measures to maintain host endogenous H_2_S within the optimal ‘anti-inflammatory and osteogenic’ range while simultaneously ‘eliminating excess pathogen-derived H_2_S’; on the other hand, real-time, non-invasive detection technologies for endogenous H_2_S concentrations are not yet mature: traditional detection methods (such as electrochemical sensors and fluorescent probes) require invasive sampling (e.g., periodontal pocket fluid, tissue biopsies) and cannot facilitate dynamic monitoring; while non-invasive detection techniques (such as near-infrared fluorescence imaging) are limited by the anatomical structure of periodontal tissues (such as gingival thickness and alveolar bone obstruction); their detection sensitivity and accuracy are difficult to predict, and they cannot provide real-time feedback for H_2_S concentration regulation. Finally, the logical relationship between H_2_S regulation strategies and traditional aetiological treatment needs to be re-examined. It must be made clear that the core aetiological root of periodontitis is the dental plaque biofilm and its pathogenic bacteria; bacteria are not only the primary source of exogenous H_2_S but also the main agents responsible for the release of inflammatory factors such as LPS. Therefore, ‘eliminating periodontal bacteria’ is the fundamental measure to interrupt the ‘bacteria–inflammation–bone deficiency’ pathological chain at the aetiological level, while H_2_S regulation is not a substitute for traditional treatment, but rather a complement and optimisation. That is, building upon traditional scaling and root planing to remove bacterial biofilms, regulating H_2_S concentrations can further repair periodontal bone damage caused by bacterial infection and inhibit the persistent effects of residual inflammatory factors, thereby achieving a synergistic therapeutic effect of ‘etiological control plus tissue repair’. If the fundamental prerequisite of bacterial removal is disregarded and reliance is placed solely on H_2_S regulation, it will be impossible to halt the ongoing destruction caused by pathogenic bacteria, nor will it be possible to prevent the repeated generation of exogenous H_2_S; ultimately, this may result in limited treatment outcomes.

In an LPS-induced osteoblast inflammation model, endogenous H_2_S levels showed an upward trend, which is thought to be associated with LPS activation of the *CSE* pathway in osteoblasts. This mechanism is consistent with reports that LPS upregulates *CSE* expression and promotes H_2_S synthesis in macrophages and chondrocytes via the Toll-like receptor 4 (TLR4)/p38 and NF-κB pathways. It is noteworthy that H_2_S, which possesses anti-inflammatory properties, actually exacerbates inflammation in this model; this may be primarily due to its concentration-dependent dual action: low concentrations of H_2_S can inhibit the NF-κB pathway to exert anti-inflammatory effects, whereas LPS-induced endogenous H_2_S may reach high concentrations of over 100 μM, thereby exacerbating the inflammatory response by upregulating signaling molecules such as NF-κB and p38. Furthermore, the method of administering H_2_S donors (e.g., in vitro incubation, intraperitoneal injection or oral administration) and the route of LPS administration (e.g., tracheal instillation, intraperitoneal injection) may influence local H_2_S concentrations and the inflammatory microenvironment; these factors need to be standardised in future studies. At present, the concentration thresholds and functional differences between bacterial-derived and endogenous H_2_S remain unclear; however, as LPS is a bacterial LPS, the increase in endogenous H_2_S it induces may produce an additive effect with H_2_S produced by the bacteria themselves at the site of infection, jointly contributing to the regulation of inflammation. Therefore, the LPS-induced upregulation of endogenous H_2_S may be a key factor in the exacerbation of inflammation in osteoblasts. Identifying its concentration threshold, regulatory pathways, and interactions with bacterial-derived H_2_S could provide new therapeutic targets for the treatment of bone inflammation-related diseases. 

In summary, in periodontitis, H_2_S plays a dual role, exhibiting both ‘pathogenic’ and ‘protective’ properties: bacterially derived H_2_S is a key factor driving the progression of inflammation and tissue damage, while endogenous H_2_S is a crucial regulatory molecule that maintains the homeostasis of periodontal tissues and participates in reparative compensation. The balance between the two directly influences the pathological course of periodontitis—when pathogenic bacteria proliferate excessively, leading to an ‘excess of exogenous H_2_S’, or when abnormal tissue cell function results in ‘insufficient endogenous H_2_S synthesis’, this balance may be disrupted, exacerbating the onset and progression of periodontitis. The dual role of H_2_S provides potential therapeutic targets for periodontitis (such as regulating endogenous H_2_S synthesis and inhibiting H_2_S production by pathogenic bacteria); however, its clinical translation requires overcoming four core challenges: ‘suitability of administration methods, differentiation of concentration sources, precise regulation and detection techniques, and synergy with conventional treatments’. Future research must establish a combined therapeutic framework ‘based on aetiological control and utilising H_2_S regulation as a restorative measure’ to fully realise the therapeutic value of H_2_S.

Taken together, the evidence from osteoporosis, fracture healing, and periodontitis suggests that the biological effects of H_2_S are highly dependent on its source, concentration, release kinetics, experimental model, and local disease microenvironment. In osteoporosis and fracture models, appropriately controlled H_2_S exposure generally promotes osteoblast and mesenchymal stem cell function, reduces oxidative stress and inflammation, and improves bone formation. In periodontitis, however, bacterially derived H_2_S in an LPS-rich, hypoxic, and acidic periodontal pocket may promote inflammation, osteoclastogenesis, and alveolar bone loss, whereas controlled host-derived or pharmacologically delivered H_2_S may exert anti-inflammatory and osteogenic effects. The apparently conflicting findings may also be related to differences between rapidly releasing donors, such as NaHS and gaseous H_2_S, and sustained-release donors, such as GYY4137, as well as differences in dose, administration route, exposure duration, and cellular or animal models.

Nevertheless, the current evidence is predominantly based on in vitro studies and small-animal models. Local free H_2_S concentrations, donor release profiles, tissue distribution, pharmacokinetics, and long-term safety have not been sufficiently defined. In addition, the effects of H_2_S on osteoclastogenesis remain context-dependent and cannot be uniformly extrapolated from periodontal models to systemic bone loss. Therefore, H_2_S should currently be considered a potential, context-dependent regulator of bone remodeling rather than an established clinical therapy. Future studies should standardize H_2_S exposure conditions, distinguish bacterial-derived, host-derived, and exogenously supplied H_2_S, establish disease-specific therapeutic concentration ranges, and evaluate controlled local or systemic delivery in clinically relevant models. For periodontitis, H_2_S-based interventions should be investigated as an adjunct to conventional biofilm control rather than as a replacement for etiological treatment.

Collectively, the evidence from osteoporosis, fracture healing, and periodontitis indicates that H_2_S has therapeutic potential only when its concentration, release kinetics, source, and local microenvironment are appropriately controlled. These requirements cannot be fully addressed by conventional fast-releasing H_2_S donors. Therefore, the next stage of translational development has focused on delivery platforms capable of providing local, sustained, and disease-responsive H_2_S release. The following section discusses H_2_S-loaded scaffolds, implant coatings, hydrogels, and pharmacological donor compounds developed to translate these biological effects into practical therapies.

## 6. Development and Application of H_2_S in Bone Deficiency Diseases

### 6.1. Bone Scaffolds Loaded with H_2_S Donors

Based on the disease-specific mechanisms discussed above, an effective H_2_S delivery platform should achieve controlled release while simultaneously matching the requirements of the local repair microenvironment. In systemic osteoporosis, H_2_S-based interventions are expected to restore the balance between bone formation and resorption. In local bone defects, periodontal lesions, and implant-associated conditions, H_2_S delivery systems should additionally support tissue regeneration, limit inflammation or infection, and maintain an appropriate local H_2_S concentration.

With rapid technological advancements, bone scaffolds have achieved significant results in the field of bone defect repair. In terms of materials, there has been an evolution from traditional metal and ceramic scaffolds to biodegradable polymers (such as polylactic acid), bioactive ceramics (such as hydroxyapatite) and composite materials, which balance mechanical support with biocompatibility and can guide new bone formation during the degradation process. In terms of function, by loading growth factors (such as osteopontin), stem cells or antimicrobial agents, they achieve a synergistic multi-functional effect of ‘support–induction of regeneration–anti-infection’, thereby addressing issues such as low repair efficiency and susceptibility to infection associated with traditional scaffolds; in terms of structural design, 3D printing technology enables personalised customisation to precisely match the morphology of the patient’s bone defect, while constructing a porous structure that mimics natural trabecular bone, facilitating cell adhesion and nutrient delivery. Currently, bone scaffolds have demonstrated promising applications in the repair of post-surgical defects following bone trauma and bone tumours, driving the evolution of bone repair from ‘replacement’ to ‘regeneration’. It is worth noting that bone scaffolds loaded with H_2_S donors have emerged as a new area of research. These H_2_S donors slowly release H_2_S as the scaffold degrades, which not only regulates osteoblast activity and promotes angiogenesis to accelerate bone regeneration, but also improves the local repair microenvironment through anti-inflammatory and antioxidant effects. This further compensates for the shortcomings of traditional scaffolds in regulating the repair microenvironment, offering a more promising new solution for bone defect repair.

In the field of bone tissue engineering, bone scaffold carriers designed to incorporate H_2_S donors have undergone continuous iteration and improvement, with a focus on ‘enhancing biocompatibility, optimising the controlled release of H_2_S, and meeting the physiological requirements of bone repair’. In an early study, silk fibroin was used as a biocompatible and biodegradable carrier for the slow-release H_2_S donor GYY4137. GYY4137-loaded porous silk fibroin scaffolds showed sustained H_2_S release and good biocompatibility, supporting the adhesion, proliferation, and viability of cultured cells [50]. H_2_S upregulated genes and proteins associated with ossification, osteogenic differentiation, bone mineral metabolism, and angiogenesis, leading to early and robust mineralization. Consequently, the GYY4137-loaded silk fibroin porous scaffold effectively supported human BMSC adhesion, proliferation, and viability [65]. With advances in material preparation techniques, the carrier form has been further refined into nanofibres—whose biomimetic fibrous structure more closely resembles the microscopic morphology of the natural bone matrix. This not only increases the loading capacity of H_2_S donors but also enables the controlled release of the donor through regulation of fibre pore size, thereby more effectively guiding the proliferation and differentiation of osteoblasts. Using polycaprolactone (PCL) and gelatin (Gel) as the polymeric matrix, a JK-2-containing nanofibre scaffold was successfully constructed via electrospinning; the H_2_S enhanced cell adhesion and proliferation [66]. Subsequently, to further enhance the functional integration of the carriers, multilayer silk nanofibre and biofilm carriers were developed; the former achieved precise control of the H_2_S release rate through the interface design of its multilayer structure. A multilayer scaffold, comprising a Poly (lactic-co-glycolic acid) (PLGA) layer containing GYY4137 sandwiched between two layers of silk nanofibres, exhibits controlled release of the H_2_S donor and supports the migration of BMMSCs onto the scaffold under serum-deprived conditions, while preventing cytotoxicity [67]. The latter, owing to its thin and flexible nature, can conform to irregular bone defect sites while also providing a certain degree of barrier function, thereby reducing external interference during the repair process. The designed scaffold exhibits excellent biofilm elimination and osteogenic capabilities, serving as an alternative treatment for implant-related bone infections. The use of gas/photothermal synergy to combat antimicrobial biofilm formation enables highly efficient sterilisation; compared with antibiotics, it offers a broader antimicrobial spectrum and, in theory, greater efficacy, although further experimental results are still required to validate this [93]. While polymeric and silk-based scaffolds are mainly intended for the regeneration of bone defects, metallic implants require a different translational approach. For implants, surface functionalization is particularly important because it can integrate local H_2_S delivery with mechanical fixation, infection control, and osseointegration. Titanium-based systems therefore represent a clinically relevant extension of H_2_S-releasing scaffold technology. In recent research, titanium-based bone scaffolds, owing to their excellent mechanical strength (capable of meeting the load-bearing requirements of bone repair), excellent chemical stability, and long-term biocompatibility, have emerged as a novel carrier for H_2_S donors. Their surfaces can be modified via coating or porous structure design to address the challenges of H_2_S donor loading and controlled release, thereby offering a solution with greater potential for clinical translation in the repair of complex bone defects (such as those resulting from severe fractures or post-surgical bone tumour defects). Titanium dioxide is deposited onto the titanium surface to serve as a reservoir for antibiotics, which is then coated with a dense layer of H_2_S donors to prevent premature leakage of the antibiotics; H_2_S is released continuously in low quantities under normal conditions, while in the presence of an infectious microenvironment, the destruction of the H_2_S donor layer by bacteria triggers a rapid and substantial release of antibiotics. The coating effectively inhibits implant-related infections, achieving an antibacterial efficacy of up to 98.7% in a rat femoral bone defect model, while reducing inflammation at the defect site and promoting osseointegration of titanium orthopaedic implants [68]. The slow-release H_2_S coating exhibits excellent biocompatibility and satisfactory hemocompatibility, significantly promotes the osteogenic differentiation of BMMSCs, and inhibits osteoclastogenesis in murine monocyte-macrophage leukaemia cells (RAW264.7 cells) [69]. In the femurs of osteoporotic rats, this coating rebalanced the peri-implant bone microenvironment, leading to accelerated bone formation and enhanced osteointegration of the titanium implants. In summary, the development of bone scaffold carriers loaded with H_2_S donors has seen a shift in material selection from natural to multi-component composites, achieving a balance between mechanical strength and bioactivity; structural design has evolved from single-component to multi-dimensional integration, with optimisation of microstructure bringing it ever closer to the hierarchical structure of the natural bone matrix, while enabling precise control of H_2_S release rates; functional positioning has shifted from passive loading to active adaptation, with the role of the scaffold evolving from a mere ‘drug container’ to a ‘regenerative microenvironment regulation platform’. This not only resolves the issue of stable H_2_S donor loading but also enables adaptation to complex clinical needs, such as irregular defects and weight-bearing bone repair, thereby forming a dynamic synergy with the physiological process of bone regeneration. In the future, efforts should be directed towards advancing bone scaffold carriers from ‘structural substitution’ towards ‘intelligent regeneration regulation’, ultimately achieving precise and efficient repair of complex bone defects.

Despite the promising results, the evidence for H_2_S-loaded scaffolds remains primarily proof-of-concept evidence. Most studies have evaluated cell adhesion, proliferation, osteogenic gene expression, or short-term bone formation, whereas fewer studies have examined mechanical performance, degradation behavior, local H_2_S concentrations, systemic exposure, or long-term inflammatory responses. The biological effects of a scaffold cannot be attributed to H_2_S alone because the carrier material, pore structure, surface chemistry, degradation products, and associated antibacterial or photothermal functions may independently influence bone regeneration. Comparisons among silk fibroin, electrospun polymeric membranes, multilayer scaffolds, and titanium coatings are therefore difficult unless H_2_S loading, release kinetics, mechanical properties, and degradation rates are standardized.

From a translational perspective, sustained and infection- or ROS-responsive release may be more clinically relevant than a single rapid H_2_S pulse. However, smart responsive systems such as multilayer scaffolds, dual-gas nanoplatforms, and ROS-responsive hydrogels may face challenges in manufacturing reproducibility, sterilization, storage stability, and regulatory classification. The current in vivo studies are also generally short-term and are conducted mainly in small-animal defect or implant models. Whether these systems can maintain therapeutic H_2_S levels in large, load-bearing defects and whether they interfere with normal host defense or vascular remodeling remain unclear.

Taken together, H_2_S-loaded scaffolds, implant coatings, and hydrogels provide promising strategies for delivering H_2_S locally and sustainably in bone defects, periodontal lesions, and implant-associated conditions. However, their therapeutic application is mainly suited to focal skeletal injuries and may not adequately address systemic bone deficiency, such as osteoporosis, which requires reproducible dosing, sufficient skeletal distribution, and broader regulation of bone remodeling. Accordingly, systemic bone deficiency may additionally benefit from pharmacological interventions with controllable exposure and activity throughout the skeleton. The development of small-molecule H_2_S-releasing compounds and multifunctional drug derivatives has therefore emerged as a complementary translational strategy, which is discussed in the following section.

### 6.2. New Type of Drug for Combating Bone Deficiency That Releases H_2_S

Currently, drugs used in the clinical treatment of osteoporosis are mainly divided into antiresorptive agents (such as the bisphosphonates alendronate sodium and zoledronic acid) and anabolic agents (such as teriparatide and romosozumab); the European Union guidelines for the treatment of osteoporosis recommend sequential therapy with these two types of drugs [6]. Antiresorptive agents act on osteoclasts; by inhibiting their activity and proliferation, they reduce the rate of bone resorption, slow down bone deficiency, and thereby maintain bone density and reduce the risk of fractures. Anabolic agents, on the other hand, tend to target osteoblasts; they activate the proliferation and differentiation of osteoblasts, promoting the formation and mineralisation of the bone matrix, and actively increasing bone mass and strength. They are particularly suitable for patients with severe bone deficiency or a high risk of fracture. Sequential therapy combines the advantages of both classes of drugs: first, anabolic agents are used to rapidly increase bone mass, followed by antiresorptive agents to maintain the therapeutic effect over the long term, thereby addressing the shortcomings of monotherapy in terms of the extent of bone mass improvement or long-term maintenance. H_2_S fundamentally addresses the shortcomings of monotherapy—namely, that it ‘can only regulate bone metabolism in one direction, requiring combination therapy to balance short-term gains with long-term maintenance’—by both inhibiting osteoclast differentiation and promoting the osteogenic differentiation of MSCs, while simultaneously enhancing bone repair potential by reversing defects in BMMSCs recruitment. Consequently, novel anti-bone deficiency drugs developed based on H_2_S possess the core advantage of achieving the dual objectives of increasing bone mass and ensuring long-term maintenance as a monotherapy. This significantly simplifies treatment regimens and enhances the sustainability of therapeutic effects, offering new possibilities for the highly effective monotherapy of osteoporosis. Currently, the treatment of osteoporosis centres on pharmacological interventions, supplemented by non-pharmacological measures. Medications include bone resorption inhibitors (such as bisphosphonates and denosumab), which suppress osteoclast activity to maintain bone density and form the mainstay of treatment; anabolic agents (such as teriparatide), which activate osteoblasts to promote bone formation and are suitable for severe cases; as well as selective oestrogen receptor modulators such as raloxifene and other drugs such as calcitonin, while calcium and vitamin D serve as basic supplements. Non-pharmacological treatments encompass exercise therapies, such as weight-bearing exercises, to enhance bone density, as well as physical therapy using modalities such as laser therapy. However, these treatments have numerous shortcomings: medications have clear limitations; bisphosphonates may cause gastrointestinal discomfort or even osteonecrosis of the jaw; teriparatide requires daily injections and treatment duration must not exceed two years; and some drugs are expensive and carry risks such as thrombosis and tumours. Exercise therapy requires long-term commitment, and its effectiveness varies significantly from person to person; the long-term efficacy of physical factor therapies lacks large-scale clinical validation. Furthermore, poor treatment adherence is widespread; patients find it difficult to persist due to drug side effects, costs, or a lack of noticeable results. Early diagnosis is also challenging, and by the time most patients suffer a fracture, their condition is already severe. Additionally, underlying medical conditions and age restrict drug selection, while monitoring methods struggle to assess efficacy accurately and promptly. Consequently, in light of the current challenges in osteoporosis treatment—including the risks of drug side effects, the limitations of non-pharmacological approaches, poor patient adherence and delays in early intervention—it is of particular importance and urgency to actively explore and develop new therapeutic drugs and innovative interventions that are safer, more effective, more convenient to use and capable of addressing different stages of the disease. This is essential for overcoming existing treatment bottlenecks, improving the overall management of the condition and better meeting patients’ clinical needs. The therapeutic effects of exogenous H_2_S treatment in preclinical animal models are both comprehensive and targeted; it not only promotes the proliferation and differentiation of osteoblasts but also inhibits the excessive activation of osteoclasts, thereby bidirectionally regulating the balance of bone metabolism. This addresses the limitations of non-pharmacological approaches (such as exercise and calcium supplementation), which can only mildly improve bone density and are unable to halt the process of bone deficiency.

The H_2_S-releasing diclofenac derivatives ACS15 and ACS32 (H_2_S-releasing diclofenac derivatives) inhibit breast cancer-induced osteoclastogenesis in vitro and prevent bone resorption in vitro; they inhibit osteoclast formation and resorption and induce osteoclast apoptosis by suppressing the IκB kinase (IKK)/NF-κB pathway and RANKL [70]. H_2_S-releasing amino-bisphosphonate DM-22, a novel H_2_S-releasing compound derived from alendronate, inhibited osteoclast differentiation and function while stimulating osteogenic differentiation in human mesenchymal stromal cells. This compound combines antiresorptive and anabolic activities and showed no significant cytotoxicity in the tested human cell models [71]. The H_2_S-releasing compound SDSS, a danshensu (DSS) derivative, reduces apoptosis in osteoblast precursor cells and enhances osteogenic differentiation by increasing antioxidant enzyme levels, reducing the activation of p38, ERK1/2, and JNK-MAPK, and stimulating the PI3K/Akt pathway [27]. A novel rhein derivative regulates bone formation and resorption and improves oestrogen-dependent bone deficiency; rhein-derived thioamide (RT) blocks RANKL-induced osteoclastogenesis by inhibiting MAPK signaling, while simultaneously promoting mineral deposition and osteogenesis [28]. The anti-osteoporosis drug clodronate offers additional benefits, such as anti-inflammatory and analgesic effects, in addition to its anti-resorptive properties. Clodronate can reverse the TNF-α-induced reduction in *CSE* expression and H_2_S production; H_2_S may be involved in the additional benefits of clodronate [32]. Natural alternatives to chemical H_2_S donors can upregulate the expression of osteogenic differentiation genes; glucoraphanin increases intracellular H_2_S levels and stimulates osteogenic differentiation in human mesenchymal stromal cells [35]. The dual-gas storage nanoplatform (UZPA-CP), constructed using upconversion nanoparticles (UCNP), can simultaneously release CO and H_2_S upon excitation by 808 nm NIR light, thereby serving as an NIR-responsive CO/H_2_S dual-gas nanogenerator. This platform can precisely regulate gas release within the osteoporotic microenvironment, synergistically exerting multiple effects, including promoting osteogenesis, inhibiting osteoclast activity, and improving the immune microenvironment. Under NIR irradiation, this gas-storage nanoplatform not only effectively optimizes the bone microenvironment but also restores the dynamic balance between osteogenesis and osteoclast activity. This comprehensive therapeutic strategy, centered on the regulation of the bone microenvironment and balancing the promotion of osteogenesis with the inhibition of osteoclast activity, offers a new approach to the treatment of metabolic bone diseases [36]. An injectable ROS-responsive H_2_S-releasing hydrogel was fabricated with a two-network configuration. The primary network utilizes sodium alginate chelated with S-arylthiooxime (SATO) and incorporates H_2_S-generating components, while the secondary network is based on photo-crosslinked Poly (ethylene glycol) diacrylate (PEGDA) [38]. By incorporating Cys-releasing microspheres capable of reacting with ROS, we have developed a composite hydrogel that exhibits superior mechanical properties and biosafety. This composite hydrogel markedly facilitates the osteogenic commitment of MSCs, regulates the polarization state of macrophages, attenuates local inflammatory reactions, and suppresses programmed cell death. The H_2_S gas released by the hydrogel reduces excessive intracellular ROS levels. The therapeutic potential of the composite hydrogel for bone defect repair and regeneration was further corroborated in vivo using an osteoporotic model. In terms of clinical translation feasibility, the advantages of this class of drugs lie in their well-defined mechanisms of action and strong ability for multi-target synergistic regulation. Furthermore, some compounds, such as ATB-346 (an H_2_S-releasing naproxen derivative) and DM-22, have already demonstrated a balance between efficacy and safety in animal models, while the low toxicity of naturally derived compounds, such as glucoraphanin and *Agaricus blazei* Murrill polysaccharide ABMP, also reduces the risks associated with clinical application. However, challenges remain: the large-scale preparation of smart delivery systems (such as UZPA-CP and ROS-responsive hydrogels) involves complex processes, requiring optimisation of production costs and stability; there is insufficient data on the in vivo pharmacokinetics and long-term safety of some drugs, necessitating validation through more systematic animal experiments and preclinical studies; furthermore, the precise regulation of H_2_S release rates and dosages remains crucial; release profiles need to be tailored to the stage of disease progression to avoid toxic reactions caused by excessive H_2_S or suboptimal efficacy resulting from insufficient dosing. In the future, interdisciplinary collaboration is required to drive the optimisation of drug formulations, deepen research into mechanisms of action, and refine clinical translation pathways, thereby facilitating breakthroughs in the clinical application of these novel H_2_S-releasing drugs for the treatment of bone metabolism disorders and inflammation-related diseases. Overall, novel H_2_S-releasing drugs show potential in preclinical studies; however, their clinical translation still faces numerous challenges that must be overcome, including safety assessment, pharmacokinetic optimisation, improvements to administration methods, and long-term efficacy monitoring.

Current treatment for periodontitis centres on ‘controlling inflammation, eliminating foci of infection, restoring function and long-term maintenance’. Basic treatment involves scaling and root planing to remove calculus and plaque; where necessary, surgical procedures such as periodontal flap surgery and guided tissue regeneration are used to address the lesions. In the later stages, loose teeth are stabilised with splints, or missing teeth are replaced with implants or dentures, while regular follow-up appointments every 3–6 months are required for maintenance. However, these treatments have significant shortcomings. Basic treatment relies on patients maintaining long-term self-discipline in plaque control; poor compliance can easily lead to recurrence. Surgical treatment has limited efficacy for severe alveolar bone resorption, is relatively invasive, involves a lengthy recovery period, and is poorly tolerated by some patients. In restorative treatment, the stability of mobile teeth is easily compromised by occlusal pressure, while options such as dental implants are costly and require a sufficient bone volume. Long-term maintenance requires frequent follow-up appointments, which some patients find difficult to adhere to; furthermore, existing treatments primarily focus on managing the condition rather than fully reversing the damage to periodontal tissues, and their suitability for patients with systemic conditions such as diabetes remains to be improved. ATB-346, an H_2_S-releasing derivative of naproxen, reduces periodontal inflammation and alveolar bone loss in rats with ligature-induced periodontitis. Compared with conventional naproxen, ATB-346 may have a lower propensity for NSAID-associated gastric mucosal damage, a potential advantage attributed to the incorporation of an H_2_S-releasing moiety [39]. LPS increases the expression of NF-κB p65, pro-inflammatory factors, and nerve growth factors in the gingiva, as well as the process of apoptosis; however, the H_2_S-releasing ketoprofen derivative ATB-352 (an H_2_S-releasing ketoprofen derivative) reduces these markers and promotes bone regeneration. Treatment with ATB-352 at a dose of 20 mg/kg significantly attenuated the inflammatory response induced by intra-gingival LPS injection and also exerted beneficial effects against bone resorption and tissue damage [46]. ABMP enhances autophagy in periodontal ligament cells, thereby reducing osteoclasts, alveolar bone resorption, inflammatory cell infiltration and gingival recession; this mechanism of action involves the H_2_S/Nuclear factor erythroid 2-related factor 2 (Nrf2) axis [47]. A redox-responsive hydrogel, fabricated from alginate and Gel and functionalized with polyphenols, integrates two key bioactive components: a conductive scaffold formed by polydopamine-coated silk microfibers decorated with poly (3, 4-ethylenedioxythiophene) (PEDOT), and bovine serum albumin (BSA)-based nanocarriers engineered for controlled H_2_S delivery [51]. Continuous H_2_S release from BSA nanoparticles recruits MSCs and drives subsequent angiogenesis and osteogenesis. Meanwhile, PEDOT-assembled polydopamine-mediated microfiber network endows the hydrogel with good conductivity, facilitating endogenous bioelectric transmission, cell alignment, and Ca^2+^ influx. This gas – bioelectric synergy further amplifies autophagy in PDLSCs and modulates macrophage polarization through lipid metabolic pathways, collectively promoting bone formation. Separately, a hyaluronic acid methacrylate-ZnO (HMZ) composite hydrogel with H_2_S-scavenging capability was developed for periodontitis treatment [53]. The HMZ hydrogel exhibits good injectability and cell compatibility, and is capable of removing H_2_S through a reaction with ZnO; The HMZ hydrogel was able to increase the cell viability of human gingival fibroblasts from 13% to 120% within 48 hours, and that of human periodontal ligament fibroblasts from 22% to 94%, while restoring mitochondrial homeostasis and alleviating inflammation mediated by the cyclic GMP-AMP synthase (cGAS)-stimulator of interferon genes (STING) signaling pathway. A 3D-printable mesenchymal stem cell–bone matrix array (MSCBM) has been engineered to recapitulate bone tissue stiffness, providing a versatile platform for assessing drug and stem cell responses in bone repair associated with chronic conditions such as osteoarthritis [55]. We have demonstrated that simulating bone stiffness in a 3D system can induce the osteogenic differentiation of cMSCs. The effects of Glutathione-conjugated garlic extract (GSGa), an H_2_S sustained-release donor (a water-soluble garlic extract bound to glutathione), on hMSCs in two-dimensional (2D) and three-dimensional (3D) cultures were investigated, revealing increased expression of the antioxidant enzyme heme oxygenase-1 (HO-1) and reduced expression of the pro-inflammatory cytokine IL-6.

The pharmacological studies reviewed here suggest that H_2_S-releasing compounds may provide a broader therapeutic profile than conventional single-target drugs by simultaneously modulating osteoclast activity, osteoblast differentiation, oxidative stress, and inflammation. However, these effects cannot be generalized across all donors or drug classes. Diclofenac-, naproxen-, ketoprofen-, bisphosphonate-, rhein-, and natural-product-derived compounds contain pharmacophores with independent biological activities; therefore, the observed effects may result from the combined action of the parent drug and H_2_S release rather than from H_2_S alone. Proper controls using the parent compound, an H_2_S-inactive analogue, and a matched H_2_S donor are required to determine the contribution of each component.

The release kinetics and pharmacokinetic behavior of these compounds are also important. A donor that releases H_2_S rapidly may be useful for acute inflammatory injury but may cause transient high exposure and systemic adverse effects. Conversely, sustained-release compounds may provide a more stable pharmacological effect but could accumulate in tissues or fail to reach effective concentrations at sites of rapid bone remodeling. In addition, most studies have been performed in vitro or in rodents, and information regarding oral bioavailability, tissue distribution, metabolism, renal clearance, drug–drug interactions, and long-term toxicity remains limited.

The translational significance of these agents is therefore promising but preliminary. Their potential advantages include dual regulation of bone formation and resorption, improved anti-inflammatory activity, and the possibility of reducing adverse effects associated with conventional NSAIDs or antiresorptive drugs. Nevertheless, clinical translation requires evidence that the compounds achieve reproducible H_2_S exposure, retain the pharmacological activity of the parent drug, and do not produce H_2_S-related toxicity or organ-specific adverse effects. Future studies should use pharmacologically relevant dosing, compare the new compounds with approved therapies, evaluate sex- and age-specific responses, and establish exposure–response relationships in large-animal and human pharmacokinetic studies.

## 7. Conclusions and Outlook

With the increasing prevalence of osteoporosis, periodontitis, and fracture-related complications, bone-loss disorders have become an important global public health challenge. This review summarizes the regulatory role of endogenous H_2_S in bone remodeling, the therapeutic effects of exogenous H_2_S in bone-related diseases, and the development of H_2_S-based scaffolds, delivery systems, and pharmacological compounds.

H_2_S is involved in multiple stages of bone remodeling and regulates the functions of osteoclasts, osteoblasts, and mesenchymal stem cells through several signaling mechanisms. Its effects on bone metabolism are not unidirectional but depend on the cellular context, concentration, exposure duration, and local microenvironment. In osteoporosis and fracture-healing models, appropriately controlled H_2_S exposure may reduce oxidative stress and inflammation, attenuate cellular senescence, promote osteogenic differentiation, and improve bone formation and mechanical strength. In periodontitis, however, the biological effects of H_2_S are more complex. Bacterially derived H_2_S in an inflammatory, hypoxic, and acidic periodontal pocket may aggravate inflammation and alveolar bone loss, whereas controlled host-derived or pharmacologically delivered H_2_S may exert anti-inflammatory, tissue-repairing, and osteogenic effects. Salivary H_2_S may also have potential as an auxiliary biomarker for periodontal disease.

Taken together, these findings indicate that the therapeutic potential of H_2_S depends critically on its source, concentration, release kinetics, and disease microenvironment. This context dependence also explains the apparently inconsistent results reported across different studies. Conventional fast-releasing donors, such as NaHS and gaseous H_2_S, may produce transient or excessive exposure, whereas sustained-release donors, H_2_S-loaded scaffolds, hydrogels, implant coatings, and other responsive delivery systems may provide more stable and disease-adapted treatment. Local delivery platforms are particularly suitable for bone defects, periodontal lesions, and implant-associated conditions. By contrast, systemic bone-loss disorders such as osteoporosis may require small-molecule H_2_S-releasing compounds or multifunctional drug derivatives with reproducible dosing, adequate skeletal distribution, and broader effects on bone remodeling. These approaches should be regarded as complementary strategies rather than replacements for conventional etiological or antiresorptive treatments.

Existing research has confirmed that, through its synergistic action via multiple targets and pathways, H_2_S effectively addresses the shortcomings of traditional therapeutic drugs—such as their unidirectional regulation of bone metabolism and significant side effects—thereby opening up new avenues for the treatment of bone deficiency disorders. Although H_2_S has demonstrated immense potential in the regulation of bone metabolism and the treatment of related diseases, its clinical translation still faces numerous challenges and areas requiring urgent breakthroughs. Firstly, the concentration-dependent effects of H_2_S represent a core scientific issue. It is necessary to further clarify the optimal concentration thresholds for endogenous and exogenous H_2_S across different disease types and stages of pathology, and to establish precise concentration control standards to avoid treatment failure or side effects caused by abnormal concentrations. Secondly, the optimisation of delivery systems is crucial. For localised diseases such as periodontitis, it is necessary to develop local delivery systems with high targeting specificity and effective controlled-release capabilities to address issues such as insufficient local concentrations and microenvironmental interference with drug efficacy that arise from systemic administration. At the same time, bone scaffolds loaded with H_2_S donors require further improvements in biocompatibility, mechanical compatibility and the synergy of degradation rates to achieve dynamic adaptation between H_2_S release and the bone regeneration process. With regard to detection technology, while existing H_2_S detection probes have already enabled the auxiliary diagnosis of periodontitis, technical bottlenecks relating to non-invasive, real-time and precise localisation still need to be overcome. There is a need to develop clinical-grade in vivo dynamic monitoring tools to provide real-time feedback for the regulation of H_2_S concentrations. Furthermore, the mechanisms underlying the synergistic effects of H_2_S with other gaseous signaling molecules (such as NO and CO), as well as the therapeutic suitability for patients with bone deficiency complicated by systemic diseases (such as diabetes and uraemia), require further investigation. 

Future research should focus on multidisciplinary integration, driving the clinical translation of H_2_S-related drugs and medical devices by optimising the structure of H_2_S donors, developing smart, responsive drug delivery systems, and defining suitable clinical patient populations and dosing regimens. At the same time, there is a need to strengthen long-term safety assessments and large-scale clinical trials to validate the efficacy and safety of H_2_S intervention strategies, ultimately providing more effective and safer treatment options for prevalent bone deficiency conditions such as osteoporosis and periodontitis, thereby alleviating the associated public health burden and economic losses.

## Figures and Tables

**Table 1:** Mechanisms and Therapeutic Strategies of H_2_S and Its Donors in Bone deficiency-Related Diseases. ↓: downregulation, ↑: upregulation.

Diseases/ Application	Subtype/Inducer/Material	H_2_S Donor/Intervention	Experimental Model and Species/Cell Type	Mechanisms and Major Biological Outcomes	Reference
Osteoporosis	Oxidative stress	NaHS	Rat model of motor-complete spinal cord injury	p38/ERK MAPK ↓; ALP, osteocalcin, collagen expression ↑; protects osteoblasts	[20]
	H_2_S deficiency	Endogenous H_2_S	*CBS*-knockout mouse model and murine BMMSCs	Regulates BMMSCs’ self-renewal and osteogenic differentiation via Ca^2+^ channel sulfhydration; *Wnt*/β-catenin ↓	[13]
	Glucocorticoid	NaHS	Dexamethasone-induced injury model in murine MC3T3-E1 osteoblasts	AMPK ↑, dexamethasone-induced osteoblast apoptosis ↓	[21]
	Uremic osteodystrophy	cinacalcet-related serum; pre-/post-haemodialysis serum	Uremic serum-treated human BMMSC osteogenic differentiation model	RANKL/OPG ratio ↓	[22]
	Postmenopausal	GYY4137	Ovariectomized estrogen-deficiency mouse model; human BMMSCs	*Wnt*16/2b/6/10b, *Wnt*/β-catenin, osteogenesis ↑	[23]
	Fracture	*CSE* overexpression	Primary rat osteoblast osteogenic differentiation/mineralization model; rat femoral fracture-healing model; HEK-293 cell model for *RUNX2* sulfhydration validation	Sulfhydrates *RUNX2* (Cys123/132); osteogenic differentiation ↑; accelerates fracture healing	[11]
Osteoporosis	Postmenopausal	*CSE*	17β-Estradiol/ERα-regulated primary human osteoblast model	Estrogen receptor α recruits to *CSE* promoter; *CSE* expression (sex-specific) ↑	[24]
	Spinal cord injury	NaHS	H_2_O_2_-induced oxidative injury model in murine MC3T3-E1 osteoblastic cells	oxidative stress, MMP ↓, *Wnt*/β-catenin ↑	[25]
	Oxidative stress	GYY4137	Murine MC3T3-E1 osteoblastic cell proliferation and osteogenic differentiation model	ERK1/2 ↓, osteoblast proliferation and differentiation↑	[26]
	Bone loss	DM-22	h-OCs and h-MSCs undergoing osteogenic differentiation	osteoclast differentiation and function ↓, MSCs ↑	[27]
	Oxidative stress	SDSS	H_2_O_2_-induced apoptosis and oxidative-injury model in murine MC3T3-E1 osteoblasts	MAPK ↓, PI3K/Akt ↑	[28]
	Ovariectomy	GYY4137	Ovariectomy-induced osteoporosis model in female rats	bone mineral density, serum calcium, phosphate, and calcitonin ↑; Parathyroid, Leptin ↓	[29]
	HHcy	Endogenous H_2_S	Hyperhomocysteinemia-induced bone-loss model in mice	Reverses HHcy-induced OPG hypermethylation; restores OPG/RANKL balance	[30]
	HHcy	Endogenous H_2_S	H_2_S-deficient, inflammatory cytokine-imbalance, and bone-homeostasis disorder model in *CBS*-deficient mice	Balances *HDAC3* and NF-κB inflammatory signaling	[31]
	Ovariectomy	RT	Ovariectomy-induced estrogen-deficiency osteoporosis model; in vitro osteoblast and osteoclast bone-remodeling models	MAPK ↓, blocks RANKL-induced osteoclastogenesis; mineralization and osteogenesis ↑	[32]
	Glucocorticoid-induced	GYY4137	Glucocorticoid-induced osteoporosis animal model	*Wnt* ligands, *Wnt*/β-catenin ↑	[33]
	Glucocorticoid-induced	GYY4137	Femoral fracture-induced calcium and phosphorus metabolism disorder model in rats	*GRα*, *CYP27B1* ↓; *CYP24A1*, calbindin-D28k and *NaPi2a* ↑	[34]
	Osteoporosis	Clodronate	In vitro inflammation-stimulated cellular model	reverses the reduction in *CSE* expression and H_2_S production induced by TNF-α	[35]
	consequences of ageing	Glucoraphanin	In vitro osteogenic differentiation model using hMSCs	mineralization and osteogenesis ↑	[36]
	BMSC senescence	Endogenous H_2_S	Bone marrow mesenchymal stem cell senescence and in vitro osteogenic differentiation models; osteoporosis animal model	S-sulfhydrates *SIRT3* (CXXC motif); stabilizes mitochondrial homeostasis; senescence ↓	[37]
	BMMSC senescence	Endogenous H_2_S	Mouse BMMSC aging and in vitro osteogenic differentiation models	Calcineurin-NFAT1 pathway ↓	[14]
	Osteoporotic microenvironment	UZPA-CP	In vitro osteogenic differentiation of BMMSC/stromal cells, osteoclast differentiation or bone-resorption models	CO/H_2_S	[38]
	Osteoporotic bone defect	SATO+PEGDA	In vitro macrophage-polarization, osteoclastogenesis, and bone-related cell-culture models	MSC osteogenic differentiation ↑, ROS ↓	[39]
Periodontitis	VSCs	NaHS	Periodontal tissue model in 8-week-old male Wistar rats	RANKL/RANK ↑	[40]
Periodontitis	Oral malodorous compounds	gaseous H_2_S	In vitro osteoblast culture exposed to H_2_S/oral malodorous sulfur compounds	ERK1/2, p38 phosphorylation ↑; osteoblast ↓	[41]
	Oral malodorous compounds	gaseous H_2_S mixed with 5% CO_2_ in air	In vitro osteoclastogenesis model	ERK1/2, p38 phosphorylation, osteoclast ↑	[42]
	Oral malodorous compounds	50 ng/mL gaseous H_2_S	In vitro osteoblast apoptosis model following H_2_S/oral malodorous compound exposure	p53, mitochondrial apoptotic pathway, caspase-8 ↑	[43]
	LPS	NaHS	Rat in vivo periodontal inflammation and osteoclastogenesis model	TLR4 signaling pathway ↑	[44]
	Nicotine and LPS	NaHS	In vitro hPDLCs model	nicotine-, p38, ERK, NF-κB ↓; osteogenesis ↑, osteoclastogenesis ↓	[45]
	Ligature	ATB-346	Ligature-induced periodontitis model in rats	NF-κB ↓, inflammation and alveolar bone deficiency, alleviate gastric mucosal damage ↓	[46]
	LPS	ATB-352	LPS-induced periodontitis model in rats	NF-κB p65, pro-inflammatory cytokines ↓	[47]
	Ligature	NaHS	Rat experimental periodontitis model	Does not cause additional bone deficiency; concurrently attenuates inflammation	[48]
	Ligature	GYY4137	Rat experimental periodontitis model	Exacerbates inflammation and bone deficiency; LC3 ↑, Beclin-1 ↑, p62 ↓	[49]
	LPS	NaHS, Cys	In vitro LPS-mediated inflammatory osteoblast model	H_2_S is predominantly produced by *CSE* in osteoblasts, upon LPS stimulation ↑	[12]
	LPS	SPRC	LPS-induced periodontitis model in rats	*CSE*/H_2_S ↑, ERK/CREB ↓, modulates Th17/Treg balance	[50]
	Ligature and LPS	ABMP	In vitro inflammatory periodontal ligament cell model; experimental animal model of periodontitis	H_2_S/NRF2 ↑, osteoclastogenesis and bone resorption ↓; periodontitis inflammation ↓	[51]
	Periodontitis with diabetes	*CTH*	Experimental periodontitis animal model under hyperglycemic conditions	NF-κB p65 ↓, reduces pro-inflammatory cytokines and bone deficiency	[52]
	Periodontitis with diabetes	BSA	Diabetes-associated periodontal bone defect/periodontal bone healing model	Gas-bioelectric coupling; MSCs, angiogenesis and osteogenesis↑; modulates autophagy and macrophage polarization	[53]
	Periodontitis	GSGa	Two-dimensional and three-dimensional human mesenchymal stem cell cultures, including the MSC bone-matrix array model	HO-1 ↑; IL-6 ↓	[54]
	Excessive H_2_S	HMZ hydrogel	Human gingival fibroblast and human periodontal ligament fibroblast models related to periodontitis	Scavenges excess H_2_S; restores mitochondrial homeostasis; cGAS-STING inflammation ↓	[55]
Periodontitis Diagnosis	Wearable hydrogel-based RF sensor	—	—	Selective AgNP-thiol binding; in situ H_2_S detection + chlorhexidine release; wireless signal transmission to mobile devices	[56]
	Fe-doped MoO_3−x_/TiO_2_ nanotube chemoresistive sensor	—	—	Fe substitution creates oxygen vacancies and defect levels; synergistic gas-sensing at room temperature and ambient light; wide range, ultra-high sensitivity, fast response	[57]
	MOMS chemiresistor	—	—	Response 1.75–5.66/5.56–12.13 at 10/100 ppm; unique-signatures for mixture identification	[58]
Periodontitis Diagnosis	NIR-enhanced colorimetric platform	—	—	NIR-enhanced peroxidase-like activity; covers pathophysiological VSC levels; smartphone-assisted non-invasive real-time monitoring	[59]
	Flexible Mo_2_CTx/MoSe_2_ heterostructure sensor	—	—	Room-temp ultrasensitive H_2_S detection; real-time breath trace analysis; distinguishes healthy vs periodontitis	[60]
	ZnO film-based LSPR sensor	—	—	Detects 0.05–3 ppm H_2_S within 10 min at 70% RH; no response to methyl mercaptan or dimethyl sulphide (excellent selectivity)	[61]
	AIE fluorescent probe (TTN)	—	—	High sensitivity and fast response; dual-signal output; real-time diagnosis + ROS generation against periodontal pathogens	[62]
Bone Defect & Regeneration	Mandibular defect	Endogenous *CSE*-H_2_S	Mandibular bone defect model and in vitro osteogenic differentiation model of BMSCs	ALP, *RUNX2*, BMSC mineralization capacity ↑	[63]
	Cranial defect	H_2_S-modified M2 exosomes	Moesin-mediated uptake of H_2_S-modified M2 macrophage-derived exosomes in a bone regeneration model.	moesin-mediated endocytosis; β-catenin, MSC osteogenic differentiation ↑	[64]
Bone Scaffolds & Implants	Silk fibroin	GYY4137 (loaded)	—	Sustained H_2_S release; supports hBMSC adhesion, proliferation, and survival; no cytotoxicity	[65]
	Silk fibroin	GYY4137 (loaded)	—	Activates osteogenic, mineralogenic, and angiogenic genes; early mineralization	[66]
	PCL + Gelatin	JK-2 (electrospun membrane)	—	H_2_S increases cell adhesion and proliferation; promotes bone regeneration	[67]
	PLLA	ZnS nanoparticles	—	Gas/photothermal synergy; biofilm elimination; broad-spectrum antibacterial; promotes osteogenesis	[68]
	Implant-associated infection	macromolecular H_2_S donor	—	Sustained low-dose H_2_S + bacteria-triggered antibiotic burst release; antibacterial efficacy 98.7%; inflammation ↓; osseointegration ↑	[69]
	Ti implants	GYY4137	—	Continuous H_2_S delivery; BMSC osteogenesis ↑; osteoclastogenesis ↓; improves osseointegration	[70]
Bone resorption (tumour)	H_2_S-releasing diclofenac derivatives	ACS15/ACS32	—	IKK/NF-κB ↓, RANKL ↓; inhibits osteoclast formation and resorption; induces osteoclast apoptosis	[71]

Abbreviation: H_2_S, Hydrogen sulfide; NaHS, Sodium hydrosulfide; ERK, Extracellular signal-regulated kinase; MAPK, Mitogen-activated protein kinase; ALP, Alkaline phosphatase; BMMSCs, Bone marrow mesenchymal stem cells; AMPK, AMP-activated protein kinase; RANKL, Receptor activator of nuclear factor-κB ligand; OPG, Osteoprotegerin; *CSE*, Cystathionine γ-lyase; *RUNX2*, Runt-related transcription factor 2; MMP, Matrix metalloproteinase; MSCs, Mesenchymal stem cells; PI3K, Phosphoinositide 3-kinase; Akt, Protein kinase B; HHcy, Hyperhomocysteinemia; *HDAC3*, Histone deacetylase 3; NF-κB, Nuclear factor kappa-B; *GRα*, Glucocorticoid receptor α; *CYP27B1*, 25-hydroxyvitamin D-1α-hydroxylase; *CYP24A1*, 24-hydroxylase; *NaPi2a*, Sodium-dependent phosphate transporter 2a; TNF-α, Tumor necrosis factor-α; CXXC, Cys-X-X-Cys; PEGDA, Poly (ethylene glycol) diacrylate; VSC, Volatile sulfur compound; ERK1/2, Extracellular signal-regulated kinase 1/2; LPS, Lipopolysaccharide; TLR4, Toll-like receptor 4; LC3, Microtubule-associated protein 1A/1B-light chain 3; Cys, Cysteine; CREB, cAMP response element-binding protein; NRF2, Nuclear factor erythroid 2-related factor 2; BSA, Bovine serum albumin; HO-1, Heme oxygenase-1; IL-6, Interleukin-6; STING, Stimulator of interferon genes; MoO_3−x_, Molybdenum trioxide; TiO_2_, Titanium dioxide; Mo_2_CTx, Molybdenum carbide MXene; MoSe_2_, Molybdenum diselenide; ZnO, Zinc oxide; LSPR, Localized surface plasmon resonance; PCL, Localized surface plasmon resonance; PLLA, Poly (L-lactic acid); ZnS, Zinc sulfide; IKK, IκB kinase.

## Data Availability

No new data were created or analyzed in this study. Data sharing is not applicable to this article.
